# Involvement of Inflammasome Components in Kidney Disease

**DOI:** 10.3390/antiox11020246

**Published:** 2022-01-27

**Authors:** Ana Karina Aranda-Rivera, Anjali Srivastava, Alfredo Cruz-Gregorio, José Pedraza-Chaverri, Shrikant R. Mulay, Alexandra Scholze

**Affiliations:** 1 Laboratory F-315, Department of Biology, Faculty of Chemistry, National Autonomous University of Mexico, Mexico City 04510, Mexico; anaaranda025@gmail.com (A.K.A.-R.); cruzgalfredo@gmail.com (A.C.-G.); pedraza@unam.mx (J.P.-C.); 2 Division of Pharmacology, CSIR-Central Drug Research Institute, Lucknow 226031, India; srivastavaanjali2324@gmail.com (A.S.); shrikantrmulay@gmail.com (S.R.M.); 3 Department of Nephrology, Odense University Hospital, Odense, Denmark, and Institute of Clinical Research, University of Southern Denmark, 5000 Odense C, Denmark

**Keywords:** acute kidney injury, chronic kidney disease, kidney transplantation, inflammasome, redox signalling, endoplasmic reticulum stress, interleukin-18, interleukin-1β, NLRP3, AIM2, caspase-8

## Abstract

Inflammasomes are multiprotein complexes with an important role in the innate immune response. Canonical activation of inflammasomes results in caspase-1 activation and maturation of cytokines interleukin-1β and -18. These cytokines can elicit their effects through receptor activation, both locally within a certain tissue and systemically. Animal models of kidney diseases have shown inflammasome involvement in inflammation, pyroptosis and fibrosis. In particular, the inflammasome component nucleotide-binding domain-like receptor family pyrin domain containing 3 (NLRP3) and related canonical mechanisms have been investigated. However, it has become increasingly clear that other inflammasome components are also of importance in kidney disease. Moreover, it is becoming obvious that the range of molecular interaction partners of inflammasome components in kidney diseases is wide. This review provides insights into these current areas of research, with special emphasis on the interaction of inflammasome components and redox signalling, endoplasmic reticulum stress, and mitochondrial function. We present our findings separately for acute kidney injury and chronic kidney disease. As we strictly divided the results into preclinical and clinical data, this review enables comparison of results from those complementary research specialities. However, it also reveals that knowledge gaps exist, especially in clinical acute kidney injury inflammasome research. Furthermore, patient comorbidities and treatments seem important drivers of inflammasome component alterations in human kidney disease.

## 1. Introduction to Kidney Disease and Inflammation in Kidney Disease

The 2020 analysis of the Global Burden of Disease Study 1990–2017 estimated the global prevalence of chronic kidney disease (CKD) to be 9.1%, corresponding to 697.5 million cases [1,2]. The all-age global prevalence of CKD increased by 29.3% during this period due to aging of the population globally. The number of deaths at all ages attributable to CKD increased by 41.5%. While age-standardized CKD mortality did not change between 1990 and 2017, it declined by 41.3% for chronic obstructive pulmonary disease, 30.4% for cardiovascular disease, and 14.9% for cancer [1,2]. Therefore, new strategies for early detection and prevention of CKD and the development of more effective therapies are needed.

CKD can result from kidney injuries of any cause if the process of injury was of sufficient duration and/or intensity. It is defined by the presence of decreased kidney function or kidney damage for more than three months, while acute kidney injury (AKI) comprises multiple renal conditions associated with a sudden decrease in kidney function. Kidney disease refers to a heterogeneous group of disorders that impact on the function or structure of the kidney. It is important to note that even milder reductions in kidney function are associated with an increased risk for AKI, complications in other organ systems than the kidneys, and mortality [3].

Inflammatory and immunological processes are intimately linked to kidney disease. They contribute to the initiation of injury in AKI, for example during sepsis, but also play an important role for AKI’s extension and maintenance phase [4], and AKI-to-CKD transition [5]. In addition, inflammatory and immunological processes are involved in a wide range of CKD causes, like diabetes mellitus or systemic lupus erythematosus. Importantly, they also contribute to CKD progression, independent of the underlying cause [6]. Finally, CKD-related morbidity, such as CKD-attributable cardiovascular disease (CVD), and CKD mortality are related to chronic inflammation [7]. 

Inflammasomes and inflammasome components have an important role in infectious and non-infectious tissue injury [8]. This review discusses the involvement of inflammasome components in the pathophysiology and course of kidney diseases. Excellent reviews have been published on the topic, reporting inflammasome taxonomy and tissue expression pattern, molecular mechanisms of inflammasome activation and function, respective renal disease models, roles in human kidney disease, inflammasome gene mutations and polymorphisms, and inflammasome-related therapeutic options, such as the Refs. [9,10,11].

In this review, we focus on up-to-date information on inflammasome component-related molecular mechanisms that contribute to the pathogenesis of kidney diseases. We put special emphasis on the interrelations between inflammasome components and redox-signalling, mitochondria, and endoplasmic reticulum stress. Furthermore, we aggregate available clinical data on inflammasome component-related gene expression, protein, and protein activity analyses, to help the assessment of inflammasome relevance in human kidney disease. Genetic polymorphisms of inflammasome components are not covered.

## 2. Introduction to Inflammasome Components and Inflammasome Biology

The inflammasome structurally contains a sensory component, a mediator of caspase-associated recruitment domains (CARD), apoptosis-associated speck-like protein containing a CARD (ASC), and procaspase-1 [10]. The inflammasome sensors are classified as nucleotide-binding domain-like receptors (NLRs), missing in melanoma 2–like receptors (ALRs), and the newly discovered pyrin, based on structural criteria [12] (Table 1). Some members of the group, such as NLRP1, NLRP3, and NLRC4, have been identified as inflammasome-forming NLRs, while others, such as NLRP6 and NLRP12, are still classified as prospective inflammasome sensors [12]. Additional molecules that could trigger caspase-1 include NLRP2, NLRP7, NLRP9, a retinoic acid-inducible gene I (RIG-I), and interferon-inducible protein 16 (IFI16), although the molecular basis is less understood [13]. A comprehensive overview of inflammasome components, their functions and activators is given in the Supplementary Table S1 online. The NLRP3-inflammasome has been widely studied and characterized. The NLRP (receptor protein) has a NACHT configuration in the centre (known as the ‘nucleotide-binding oligomerization domain (NOD)’), an LRR domain (leucine-rich repeat) at the C terminus, along with a CARD domain/Pyrin Domain (PYD) at the amino-terminal end. The ASC protein (a mixture of PYD and CARD) may bind with NLRP3′s amino-terminal pyrin domain thus stimulating procaspase-1 [10]. NLRP3, ASC and procaspase-1 form a multiprotein inflammasome complex that activates caspase-1. Active caspase-1 converts pro-interleukin-1β (pro-IL-1β) and pro-interleukin-18 (IL-18) to mature IL-1β and IL-18 [14]. Production of mature IL-1β and IL-18 involves two distinct mechanisms: the production of pro-IL-1β and pro-IL-18 by nuclear factor κ-light-chain-enhancer of activated B cells (NF-κB) signalling (termed “signal 1”) and their cleavage by caspase-1 to generate mature IL-1β and IL-18 (termed “signal 2”) [14]. Signal 1 regulates the expression of numerous inflammasome components as well as the synthesis of “pro” forms of cytokines, even though the NLRP3 inflammasome complex is only important in signal 2. Pathogen-associated molecular patterns (PAMPs) and danger-associated molecular patterns (DAMPs) activate the toll-like receptor (TLR)-mediated NF-κB signalling to provide signal 1, whereas various metabolically and environmentally formed crystal particles and adenosine triphosphate (ATP) provide signal 2 for activating the inflammasome complex [15]. These stimuli that activate the inflammasome are different from the ones that provide signal 1. As a result, the inflammasome acts as a detector, and its induction is a frequent way for cells to react to cellular stress. 

Interestingly, NLRP3 signalling during chronic tissue remodelling does not necessarily involve the inflammasome complex. NLRP3 expression in epithelial cells affects tissue remodelling following chronic trauma in parallel with cell death signalling. For example, NLRP3 enhanced angiogenesis in mice, independent of IL-1β, during cutaneous wound healing [16]. Moreover, an NLRP3 augmented transforming growth factor β (TGFβ)/Smad signalling pathway independent of inflammasome leads to tubular-interstitial fibrosis during development of CKD [9,17]. Furthermore, similar to epithelial cells, it was found that an NLRP3-augmented TGFβ-Smad pathway in kidney fibroblasts independent of the inflammasome complex contributed to kidney fibrosis [18].

The diversity reported in NLRP3 activation highlights the inflammasome’s potential role as a detector of stress/injury in cells that operates as a unifying mechanism [19,20]. Three main mechanisms have been proposed to activate the NLRP3 inflammasome: cathepsin B release following lysosomal injury, potassium efflux, and phagocytosis [21,22]. Canonical inflammasome activation signalling involving caspase-1 has been extensively studied. The mechanisms involved in non-canonical inflammasome activation signalling via caspase-11 or other effectors, such as caspase-4, caspase-5, and caspase-8, are still incompletely understood [19,23].

In addition, post-translational protein modifications (PTMs) contribute to the regulation of inflammasome components’ activity and degradation. Such PTMs have been reported for inflammasomes’ sensory and ASC components, and include phosphorylation, ubiquitination, deubiquitination, S-nitrosylation, and SUMOylation [24,25]. Bruton tyrosine kinase interacts with both ASC and NLRP3 through kinase domains. This is thought to promote activation of the NLRP3 inflammasome. Therefore, the bruton tyrosine kinase inhibitor ibrutinib was recently tested in two kidney disease models, but the results for this substance were not promising [26]. While PTMs are frequently reported in kidney disease [27], the specific PTMs of inflammasome components in kidney disease have not been established yet, and further research is indispensable.

**Table 1:** Proposed inflammasome components and molecular mechanisms related to their function and activation. The table gives the components most consistently reported in kidney disease to date.

Inflammasome Component	Structure	Function	Activator	Reference
NLRP3	Consists of three components-NLRP3 scaffold, PYCARD adaptor (ASC), which functions as a caspase-1 activator, and caspase-1.	Initiates an inflammatory form of cell death and triggers the release of proinflammatory IL-1β and IL-18.	Bacterial and viral nucleic acids, LPS, and damage-associated molecular patterns, such as ATP, uric acid, and amyloid β peptides. Ionic flux, mitochondrial dysfunction, and the production of reactive oxygen species, and lysosomal damage have been shown to trigger its activation.	[12,21,22]
NLRP6	Composed of three domains. The N-terminus consists of a pyrin domain (PYD) and is considered the essential element for inflammasome assembly as it interacts with ASC. The NBD builds the central module of NLRP6 and is followed by the C-terminal LRR domain, which senses DAMPs and MAMPs.	Regulates the production of IL-18	Bacterial products, bacterial acylated lipopeptides	[28,29,30]
NLRC4	NLRC4 contains a common three-domain structure: An N-terminal homotypic interaction domain, a central nucleotide-binding domain, and a series of C-terminal LRRs.	The activation of NAIP proteins attracts and activates NLRC4, which in turn attracts caspase-1 either directly or indirectly through ASC, causing inflammatory responses	Bacterium’s type 3 secretion system (T3SS) and flagellin	[31,32]
NLRC5	Largest NLR family member, consisting of 1 866 aa with C-terminal 27 LRRs. Sequence analysis suggests that NLRC5 is most similar to CIITA among NLR family members.	Regulation of MHC class I gene expression, inflammasome activation in response to bacterial infection through a mechanism involving heterodimerization with NLRP3.	Bacterial PAMPs and crystals	[33,34,35]
AIM2	AIM2 consists of two domains connected through a long linker: an N-terminal PYD domain, and a C-terminal HIN-200 domain. HIN-200 region directly binds to DNA while the PYD region mediates protein-protein interaction.	Triggers the formation of inflammasomes that also contain ASC and caspase-1, and that induce the cleavage of caspase-1, the maturation ofIL-1β, IL-18, and pyroptosis.	dsDNA, exogenous DNA of bacteria (e.g., *Listeria monocytogenes*) and viruses (e.g., *Papillomavirus*), as well as endogenous DNA.	[36,37,38,39]
Pyrin	Pyrin is coded by the *MEFV* gene, and its mature form includes a PYD, two B-boxes, and a coiled-coil domain.	Causes various modifications (glycosylation, adenylation, ADP-ribosylation, etc.) of Rho GTPases, causing the rearrangement of the cytoskeleton and subsequent activation of pyrin inflammasomes.	*Clostridium difficile* TcdB, *Clostridium botulinum* C3, and *Vibrio parahaemolyticus* VopS proteins.	[40,41]
ASC	Two death domains (pyrin and CARD). ASC interacts with cell death executioners.	ASC is a central adaptor molecule of the inflammasome complex, *activation* of caspase-1, mediates the secretion ofIL-1β and IL-18.	Activation of NLRP3 protein recruits ASC.	[42,43]
Caspase-1	Active Caspase-1 contains two heterodimers of p20 and p10. It contains a catalytic domain with an active site that spans both the p20 and p10 subunits, as well as a noncatalytic CARD.	Activated Caspase-1 proteolytically cleaves pro IL-1β and pro-IL-18 into their active forms, IL-1β and IL-18. The active cytokines lead to a downstream inflammatory response. It also cleaves Gasdermin D into its active form, which leads to pyroptosis.	Autoactivates when it is assembled into the filamentous inflammasome complex by autoproteolysis into the p10 and p20 subunits.	[44,45]

NBD—Nucleotide-binding domain; LRR—leucine rich repeat; CARD—caspase activation and recruitment domains; PYCARD—PYD And CARD Domain Containing; CASP1—caspase-1; IL—interleukin; ASC—apoptosis-associated speck-like protein containing a CARD; DAMPs—damage-associated molecular patterns; MAMPs—microbe- or pathogen-associated molecular patterns; NLR—NOD-like receptor; NFκB—nuclear factor κ-light-chain-enhancer of activated B cells; MHC—major histocompatibility complex; NAIP—neuronal apoptosis inhibitory protein; CIITA—class II transactivator; AIM2—Absent In Melanoma 2; HIN—Hematopoietic expression, interferon-inducible nature, and nuclear localization; MEFV—mediterranean fever; CGAS—Cyclic GMP-AMP synthase; LPS—lipopolysaccharide; ATP—adenosine triphosphate, NLRC—NLR family CARD domain-containing protein.

## 3. Inflammasome Components in AKI

### 3.1. Preclinical Data on Inflammasome Components in AKI

AKI is associated with a high incidence of cell death and the production of cellular debris. The most prominent cause of acute renal damage is acute tubular necrosis (ATN). ATN is linked to an inflammatory response comprising monocytes/macrophages and neutrophil infiltration, which exacerbates the kidney damage [46,47]. The evidence for the inflammasome’s function in acute renal disease is compelling. Deficiency of the inflammasome component, caspase-1, in animals provides resistance against AKI in several models such as cisplatin and ischemia-induced acute renal failure [48,49,50,51]. Moreover, inhibition of NLRP3 by hydroxychloroquine decreased NF-κB signalling, as well as cathepsin-B and L activities, and protected rodents from AKI [52]. The extracellular matrix (ECM) components biglycan and hyaluronan, as well as ATP acting via P2X7 receptors, activated the NLRP3 inflammasome [53,54]. The pathogenic roles of the NLRP3 inflammasome have been demonstrated in ischemia-reperfusion injury (IRI) [52,55,56,57], folic acid-induced AKI [58], rhabdomyolysis-induced kidney injury [59], and contrast-induced kidney injury [60]. During contrast-induced AKI, canonical NLRP3 inflammasome activation in local and migratory macrophages led to elevated IL-1β levels in mice, whereas *Nlrp3*-/- animals were protected [60]. Infection-induced AKI models have also been shown to activate canonical inflammasomes. In a caecal ligation puncture model (sepsis-induced AKI), NLRP3 deficiency and caspase-1 suppression reduced kidney injury, inflammation, and caspase-1 activation [61]. Furthermore, mice with caspase-1 deficiency were protected from endotoxemic AKI, hypotension, and mortality caused by LPS [62]. Neutralization of IL-1β and IL-18, on the other hand, was unable to reverse LPS-induced AKI, implying that the non-canonical inflammasome and pyroptosis play an important role [63]. Accordingly, the induction of pyroptosis has been related to caspase 11 upregulation, the non-canonical pyroptosis pathway, demonstrated in renal proximal tubular cells treated with LPS. The authors suggest that pyroptosis induction could be an early event in septic models [64]. Furthermore, Astragaloside-IV protected from cisplatin-induced AKI by promoting autophagy and inhibiting NF-kB signalling, thus lowering the expression of inflammasome components [65]. Interestingly, NLRP1 activation was elevated in cisplatin-induced AKI, likely upstream of caspase-1 activation [66]. In this model, the deletion of caspase 11 promotes the downregulation of IL-18 urine secretion, decreasing tubular damage, immune macrophage, and neutrophil infiltration, and attenuating renal dysfunction. On the other way, caspase 11 upregulation induces the cleavage of gasdermin D into gasdemin N to trigger pyroptosis [67]. In addition, the deletion of GASMDE, a member of the GASDM family, decreases cisplatin-induced damage by blocking pyroptosis and IL-1β release [68]. During the pathogenesis of uric acid-induced nephropathy, uric acid crystals activate the NLRP3 inflammasome, suggesting a novel pathomechanism of crystalline nephropathy [69]. The NLRP3 inflammasome complex must be activated for renal IL-17A to be produced, which is an essential proinflammatory cytokine in AKI [70]. The discovery of the underlying mechanisms could assist the therapeutic suppression of IL-17A in AKI. Further, Deplano et al. reported that P2X7R (P2X purinoceptor) deficiency in rats reduced the activation of NLRP3-inflammasome in macrophages, and also crescentic glomerular damage in experimental nephrotoxic nephritis coupled with crescentic glomerulonephritis [71]. Together, these data suggest that inflammasome components can be used as therapeutic targets for treatment of AKI.

Several of the signalling molecules involved in regulating programmed cell death also modulate inflammasome activation in a cell-intrinsic manner. Necroptosis is typically seen as a back-up that kicks in when apoptosis is prevented; pyroptosis is a fundamental cellular mechanism triggered by the inflammasome in response to a wide spectrum of PAMPs and DAMPs [72]. Activation of inflammatory caspases such as caspase-1, caspase-4, caspase-5, and caspase-11 leads to pyroptosis, which relies on gasdermin-D to produce plasma membrane pores [73]. Due to their limited potential to release IL-1β, the prevalence of pyroptosis in tubular epithelial cells (TECs) has been disputed [74]. Pyroptosis, characterised by elevated caspase-1 activation and IL-1β production, has been proposed to emerge in kidney tubular cells during renal IRI [75].

Mice lacking distinct inflammasome components were utilised to establish the inflammasome’s participation in several experimental models of renal damage, but the specific role of intrinsic renal cells in inflammasome activation remains unknown [76]. Some studies stated that TEC apoptosis and pyroptosis are the key drivers of contrast-induced AKI [77,78], others did not show TEC apoptosis [60,79], canonical inflammasome formation in TECs, or IL-1β release from TECs in response to contrast-induced AKI [60,80]. Necroptosis mediated NLRP3 inflammasome plays a key role in the pathogenesis of lupus nephritis [81] as well as the transition from AKI to CKD [82]. Emerging evidence indicates that several signalling mechanisms that had been assumed to be biochemically independent for a long time communicate with one another. Nevertheless, the impact of apoptotic and regulated necrosis signalling molecules on the inflammasome is inconsistent and depends on the cell type and cellular environment. As a result, we are still a long way from understanding how these chemicals lead to altered inflammasome expression across various settings, as well as why these cell death mechanisms have developed to participate in inflammasome activation.

#### 3.1.1. Preclinical Data on Redox Signalling and Inflammasome Components in AKI

Inflammasome activation via reactive oxygen species (ROS) has been fully reported upstream of NLRP3 priming. The latter has been demonstrated by ROS inhibitors that blocked the priming steps of inflammasome [83,84]. Additionally, it has been hypothesized that DAMPs and PAMPs enhance ROS generation by interacting with pattern recognition receptors, inducing inflammasome activation. In IRI-induced AKI, the renal damage produces endogenous DAMPS, heat shock protein and ATP, which prompts ROS and activates inflammasome to cause tubular necrosis [55]. Moreover, in vitro, the treatment of human kidney proximal epithelial (HK-2) cells with hydrogen peroxide (H_2_O_2_), a ROS, activates NLRP3 [85].

Nicotinamide adenine dinucleotide phosphate (NADPH) oxidases (NOXs) and mitochondria are the principal ROS sources that induce inflammasome activation [86,87,88,89]. NOXs are a family of seven members, of which NOX1, NOX2, NOX4 and NOX5 are expressed in renal cells [90]. The production of radical anion superoxide (O_2_^•−^) by NOXs requires the assembly of p22- and glycoprotein 91 (gp91)-phox membrane subunits with the cytoplasmic subunits p47-, p67-phox, and ras-related C3 botulinum toxin substrate 1 (Rac1) [91]. In macrophages, using diphenyleneiodonium (DPI), a NOXs blocker, inhibits the proteolytic excision of caspase-1, impeding inflammasome activation [92]. The levels of NOXs are commonly elevated in AKI models, leading to ROS overproduction and then inflammasome activation [93]. NOXs activation also promotes the upregulation of redox signalling, such as NF-κB and activating protein-1 (AP-1) via mitogen-activated protein kinases (MAPK), which are involved in the priming steps of inflammasome activation [94]. Moreover, ROS induces NF-κB activation through degradation of its inhibitor, IκBα, releasing and permitting NF-κB translocation to the nucleus to induce the expression of inflammatory genes, including NLRP3 [95,96,97]. 

Liu et al. [98] showed that death-associated protein kinase (DAPK) was implicated in NLRP3 activation via ROS overproduction in an AKI model induced by paraquat (PQ) (Table 2) [98]. PQ is an herbicide used in agricultural production that produces ROS, involving cyclic reactions of reduction/oxidation, which ultimately activates NF-κB and DAPK. PQ also upregulates the phosphorylation of IκBα, which induces nuclear translocation of NF-κB, the activation of DAPK, and consequently, NLRP3 induction. The use of BAY, an inhibitor of NF-κB, attenuated this NLRP3 induction [98]. This group also showed that the DAPK inhibitor impedes caspase-1 activity, but it could not attenuate NLRP3, suggesting that DAPK protein is not required to priming steps of inflammasome but for activation. Moreover, Mahmoud et al. [99] reported that the activation of peroxisome proliferator-activated receptor-gamma (PPARγ) by ferulic acid mitigates inflammation through prevention of NLRP3 activation via NF-κB inhibition and nuclear factor erythroid 2-related factor 2 (Nrf2) activation in a methotrexate-induced nephrotoxicity model. In this model, the activation of Nrf2 and PPARγ prevents the upregulation of NF-κB, suggesting that the activation of these proteins decreases ROS, preventing NLRP3 priming. In summary, the generation of ROS in AKI models promotes NLRP3 priming by reducing antioxidant response and augmenting the inflammatory response that triggers NLRP3 assembly. 

#### 3.1.2. Preclinical Data on Mitochondria and Inflammasome Components in AKI

Mitochondria are essential in the kidney because they produce the energy, in ATP form, required for tubular reabsorption, principally in the proximal tubules (PT) [101]. Mitochondrial disruption commonly leads to inflammasome activation through mitochondrial ROS (mtROS) production [102,103]. Following this, the use of antioxidants that target mitochondria like (2-(2,2,6,6-Tetramethylpiperidin-1-oxyl-4-ylamino)-2-oxoethyl)triphenylphosphonium chloride (mitoTEMPO), N-acetyl cysteine (NAC), and resveratrol can prevent inflammasome activation [89,103,104]. Additionally, the use of rotenone, an inhibitor of complex I, has been shown to inhibit NLPR3 by reducing mtROS and mitochondrial dysfunction [105]. Moreover, IL-22, an interleukin that reduces loss of membrane potential and avoids mtROS production, impeded NLRP3 activation in the acetaminophen-induced AKI model [102].

Mitochondrial proteins have been implicated in inflammasome activation via mtROS [89,106]. Excessive mtROS promotes NLRP3-thioredoxin-interacting protein (TXNIP) translocation from cytosol to the mitochondria, where TXNIP interacts with mitochondrial thioredoxin 2 (TRX2), inhibiting TRX2 activity. TXNIP is then released and directly binds to leucine-rich regions of NLRP3, leading to inflammasome formation [106]. During IRI, mtROS trigger the interaction between TXNIP and NLRP3 three days after reperfusion, promoting renal injury [89]. In addition, mitochondrial antiviral signalling protein (MAVS) is an adaptor protein involved in the relocalization and association of NLRP3 to the mitochondria, facilitating its oligomerization with caspase-1 and ASC [88]. 

Proteins participating in mitochondrial dynamics also activate the inflammasome. Mitochondrial dynamic comprises the balance between mitochondrial fission and fusion, which requires the recruitment of proteins to the mitochondria that carry out fission or fusion [107,108]. Moreover, under ROS production, the mitochondrial fission protein dynamic related protein 1 (DRP1) is recruited to the outer mitochondrial membrane to trigger fission. However, excessive mitochondrial fission impairs mitochondrial function, causing further mtROS production and inflammasome activation [109]. In accordance with this, Liu et al. [110] found that DRP-1 promotes NLRP3 activation during sepsis-induced AKI, triggered by LPS, highlighting the involvement of mitochondrial proteins in the promotion of inflammation via NLRP3. Supporting this, the use of mitochondrial division inhibitor 1 (Mdivi-1), a DRP1 inhibitor, decreases the inflammasome components (i.e., NLRP3, caspase-1, IL-1β and IL-18) [110]. Furthermore, the upregulation of DRP-1 leads to mitophagy induction, which is a negative regulator of inflammasome; but, defective mitophagy is commonly found in AKI, contributing to mtROS accumulation [111,112,113]. In line with this, in contrast-induced AKI, the contrast agent iohexol increases NLRP3 and malondialdehyde (MDA) (a biomarker of oxidative stress) levels and decreases the antioxidant activity of superoxide dismutase (SOD) and the protein levels of phosphatase and tensin homolog (PTEN)-induced putative kinase 1 (PINK1), Parkin and microtubule-associated proteins 1A/1B light chain 3B (LC3-II) [114]. Likewise, the autophagy inhibitor 3-methyladenine (3-MA) promotes inflammasome activation [113]. Furthermore, in the contrast-induced AKI model, the activation of mitophagy by PINK1/Parkin prevents NLRP3 activation, decreasing kidney injury by avoiding apoptosis in renal tubular epithelial cells [97]. Besides, the lowering of sirtuin 1 (SIRT1) and 3 (SIRT3), involved in mitophagy, promote inflammasome activation in AKI models [85,115,116]. SIRT1 and SIRT3 are NAD-dependent deacetylases that protect against mitochondrial dysfunction by avoiding mtROS, and their deregulation has been associated with mitophagy deregulation, leading to inflammasome activation [115,117]. Gao et al. [117] reported that in sepsis induced by caecal ligation and puncture (CLP), the blocking of mitophagy upregulates NLRP3, ASC, caspase-1 and IL-1β. In addition, the inhibition of SIRT1 with EX527 blocked Parkin translocation to the mitochondria, suggesting that SIRT1 is required for mitophagy activation, impeding inflammasome stimulation [80]. In the CLP model, a perforation in the cecum is realized, which permits fecal material release into the peritoneum, generating an exacerbated immune response, including NLRP3 inflammasome activation [118]. Therefore, mitophagy is required to avoid excessive NLRP3 activation in this model. 

In addition to mitophagy, crosstalk between inflammasome and intrinsic apoptosis has been found in leucocytes, involving caspase-8 and caspase-1 [119,120]. In this context, in IRI-induced AKI, NLRP3 modulates a non-canonical caspase-8-activating platform at the mitochondria, required for epithelial cell death. Although caspase-8 activation via NLRP3 is independent of canonical NLRP3 activation, it involves ASC, suggesting that caspase-8 is a downstream protein of NLRP3 [80].

In summary, mitochondria are involved in inflammasome activation in AKI models, related to mtROS production. The latter activates the proteins TXNIP and MAVS, imported to the mitochondria to induce inflammasome assembly by interacting with NLRP3. On the other hand, mitophagy is considered an inflammasome inhibitor, since its blocking activates NLRP3 components. 

#### 3.1.3. Preclinical Data on Endoplasmic Reticulum Stress and Inflammasome Components in AKI

Endoplasmic reticulum-stress induces inflammasome activation by triggering unfolded protein response (UPR) [121]. The three pathways of ER stress have been implicated in inflammasome activation: pancreatic eukaryotic translation initiation factor 2a (eIF2a) kinase (PERK), inositol-requiring protein 1 (IRE1α) and activating transcription factor 6 (ATF6) by promoting caspase-1 activation and IL-1β maturation [122]. In vitro, the three signalling pathways induce UPR activation in cadmium-induced nephrotoxicity, leading to NLRP3 activation [123]. In addition, the activation of SIRT1 avoids these effects by generating the deacetylation of X-box binding protein-1 (XBP-1), a member of IRE-1α, into its spliced form XBP-1s, suggesting that SIRT1 is required to avoid inflammasome activation mediated by ER stress [123]. Guo et al. [124] reported that the use of taurine-conjugated ursodeoxycholic acid, a chemical chaperone that alleviates ER stress, prevents inflammasome activation in aldosterone-induced AKI. Moreover, resting NLRP3 localizes in the ER; meanwhile, ASC and NLRP3 localize in perinuclear membranes of ER and mitochondria under inflammasome activation [113,125]. These works suggest that ER is crucial in inflammasome activation. Indeed, Wang et al. [125] demonstrated that HK-2 cells stimulated with angiotensin II (Ang II) showed inflammasome activation and ER-stress induction and the pretreatment with 4-PBA, an ER-stress inhibitor, decreases ASC and NLRP3 [125]. It has been proposed that ER-stress-induced inflammasome activation is mediated by ROS overproduction. Following the latter, in rhabdomyolysis-induced AKI, the treatment with the antioxidant anisodamine decreases inositol-requiring enzyme-1α (IRE-1α), CCAAT/enhancer-binding protein (C/EBP) homologous protein (CHOP) and activating transcription factor 4 (ATF4), reducing NLRP3 inflammasome components (i.e., NLRP3, caspase-1, IL-1β and IL-18) [126], suggesting that ROS is implicated in ER-stress protein’s activation. The authors also showed that inflammasome activation induced via ER stress depends on the interaction between NLRP3 and TXNIP [126]. Thus, these results highlight that activation of the inflammasome by mitochondria and ER stress are intimately related in AKI models.

**Figure 1:**
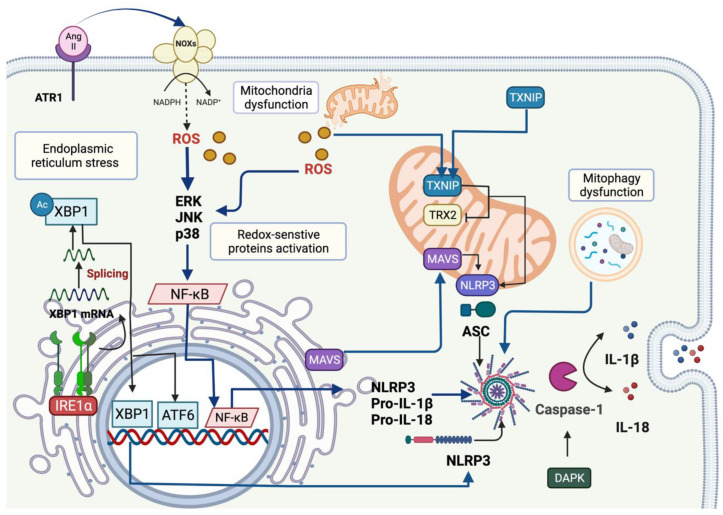
Activation of NOD-like receptor (NLR) family pyrin domain containing 3 (NLRP3) inflammasome mediated by redox-signalling, mitochondria and ER-stress during acute kidney injury (AKI). The binding of angiotensin II (Ang II) to its receptor, the angiotensin receptor 1 (ATR1), triggers ROS overproduction by stimulating NADPH oxidases (NOXs) in the plasmatic membrane. The ROS produced by NOXs induces the activation of the redox-sensitive pathway, including extracellular signal-regulated kinase (ERK), c-Jun N-terminal kinases (JNK) and p38, proteins able to activate to nuclear factor κ-light-chain-enhancer of activated B cells (NF-κB). The latter promotes the transcription of target genes such as NLRP3, pro-interleukin 1 β (IL-1β) and pro-interleukin 18 (IL-18). Under mitochondrial dysfunction, ROS are also generated, named mitochondrial ROS (mtROS), which activates the priming steps of inflammasome mediated by the redox-sensitive proteins. Further, excessive mtROS leads to the translocation of thioredoxin-interacting protein (TXNIP) from the cytosol to the mitochondria. In mitochondria, TXNIP interacts with thioredoxin 2 (TRX2), inhibiting its activity, promoting the interaction with NLRP3 protein. Moreover, mitochondrial antiviral signalling protein (MAVS), an adaptor protein, is involved in NLRP3 localization in the mitochondria and participates in the inflammasome formation by promoting this interaction with apoptosis-associated speck-like protein containing a CARD (ASC). High levels of mtROS activate mitophagy, a negative regulator of inflammasome; however, in the case of AKI, mitophagy is dysfunctional, contributing to NLRP3 inflammasome activation. Another protein that activates inflammasome is death-associated protein kinase (DAPK), which activates caspase-1. On the other hand, the endoplasmic stress reticulum produces ROS and triggers unfolded protein response (UPR), recognized as an inductor of the inflammasome. Figure created using BioRender (Toronto, ON, Canada).

Figure 1 summarizes the mechanisms that induce NLRP3 inflammasome activation mediated by redox signalling, mitochondria and ER stress in renal epithelial cells of AKI models.

#### 3.1.4. Preface to Clinical Data Analyses of Inflammasome Components in Kidney Disease

In 2010, it was shown that the gene expression of NLRP3 was significantly increased in nondiabetic patients with different causes of acute and chronic kidney diseases. These analyses included renal tissue samples from patients with IgA nephropathy and membranous glomerulonephritis, acute tubular necrosis and crescentic glomerulonephritis, and also hypertensive/vascular nephrosclerosis [127]. These data illustrate the potentially widespread involvement of inflammasome components in kidney disease. However, they also highlight the complexity in studying inflammasome components in human kidney disease, as hypertension, diabetes, and other comorbidities are frequent. Additional points of caution can be raised. As in other areas of complex regulations in kidney disease, the combined investigation of a set of meaningful molecular parameters is inevitable. Thorough analyses of gene expression, protein characteristics, and protein activity together enable a better understanding of molecular networks [128]. Ideally, in investigations of inflammasomes, this comprises the gene expression and protein amount of inflammasome-related components, post-translational modifications of the inflammasome sensory components, inflammasome assembly and proteolytic activity, and production of mature interleukins [11]. On the other hand, the spectrum of relevant molecular partners for inflammasome components is wide. It not only includes molecules involved in canonical and non-canonical inflammasome-related mechanisms, but also inflammasome-independent mechanisms of sensory inflammasome components, as repeatedly shown for NLRP3 [10,11]. In addition, intensive molecular links exist for different forms of regulated cell death [8,129] and a close and bi-directional relation to caspase-8 has been reported [11,130]. Caspase-8 was therefore included in our search of clinical data in relation to inflammasome components.

The current review, and a great number of original research articles, report analyses of IL-1β and IL-18 to propose an underlying action or activation of inflammasome components. The creation of functional IL-1β and IL-18 through canonical inflammasome activity has been described in the introduction, and caspase-8-mediated maturation of IL-1β through non-canonical inflammasome activation has been reported [11]. Besides, inflammasome-independent maturation of Il-1β and IL-18 occurs, for example, through neutrophil-derived serine proteases or proteases originating from microorganisms [131]. As a rule of thumb, inflammasome-dependent interleukin processing will be of importance if the involved inflammatory cells are of the monocytes/macrophages lineage. This brings us to the last critical issue of inflammasome component involvement in kidney disease. Interpretation of the data is compartment- and context-dependent. The context in this respect can be a comorbidity, like diabetes mellitus [132], or higher age [133], which themselves influence inflammasome components, and therefore need to be accounted for by matched control groups. The context also includes the state of uraemia in a patient with kidney disease. The uremic toxin indoxyl sulfate, for example, can decrease NLRP3 [134]. Thereby, the degree of renal function impairment, such as GFR, will interfere with the observed NLRP3 regulation in a given kidney disease, and needs to be accounted for in the analyses. The compartment in which inflammasome components are analysed is likewise important for the interpretation. Circulating immune cells are involved in the pathophysiology of several kidney diseases [135], but they are also influenced by uremic conditions [136] and contribute to CKD-dependent morbidity [7]. In the kidney, the mechanisms involving inflammasome components partially differ between resident and recruited immune cells on the one hand, and cells of tubules or glomeruli on the other [11]. In addition, inflammasome-related characteristics like mode of activation and expression level of the components change during the differentiation from monocytes to macrophages or dendritic cells [137,138]. Furthermore, even distant events and organ crosstalk need to be considered. A study of kidney function after liver transplantation suggested a relation between the IRI of the liver transplant and AKI of the organ recipient, involving liver and circulating IL-18 [139]. Not least, the interpretation of inflammasome components in urine requires consideration of several aspects. For example, IL-18 protein was reported to be expressed in intercalated cells from the late distale tubule to the collecting duct of the kidney [140] and tubular epithelial cells [141], and IL-18 protein expression was inducible in glomeruli in Lupus nephritis [142]. Currently, IL-18 is interpreted as a marker of tubule injury [143]. Increased concentrations in the urine could result from renal cell damage, leaderless secretion, or decreased or insufficient tubular reuptake after glomerular filtration, the latter in analogy to IL-2 [144]. Interleukin-18 can predict AKI, but it is not a marker for AKI severity or kidney recovery [145]. This suggests that IL-18 found in urine, mirrors regulated cellular processes rather than just passive loss due to cell damage. While IL-18 is mainly regarded as a proinflammatory cytokine, its role in homeostasis and protection of epithelial and mucosal surfaces has also been stressed [146]. 

Resulting from all the considerations above, we chose an inclusive approach for our analyses of inflammasome components and inflammasome substrates that have been reported in the clinical research of kidney disease.

### 3.2. Clinical Data on Inflammasome Components in AKI

Acute kidney injury refers to a sudden loss of kidney function. It can be defined and staged according to the KDIGO guidelines using serum creatinine increase within the prior seven days and/or the presence of oliguria [147]. AKI is a heterogeneous syndrome with a broad range of etiologies. Therefore, the following section summarizes the clinical data on inflammasome components according to the respective AKI cause. It should be noted that in clinical practice, pathophysiological mechanisms in AKI may overlap, thereby complicating the dissection of underlying molecular mechanisms [148].

#### 3.2.1. Cardiac Surgery-Associated AKI (CSA-AKI)

The pathophysiology of CSA-AKI includes mechanisms related to IRI, significant alterations in hemodynamics, oxidative stress, and the activation of inflammatory processes [149]. The use of cardiopulmonary bypass (CPB) leads to the activation of proinflammatory pathways, the coagulation cascade and the complement system. No data on inflammasome components in human CSA-AKI have been published so far. A current trial (NCT04125069) is investigating the relation between preoperative mitochondrial dysfunction, which may contribute to inflammasome activation, and CSA-AKI. In preclinical studies on cardiac transplantation, IRI resulted in the release of RNA [150], and the release of mitochondrial DNA (mtDNA) into human plasma during cardiopulmonary bypass surgery has been reported [151]. Mitochondrial DNA, and especially oxidised mtDNA, which is formed in the presence of ROS, can activate the NLRP3 inflammasome and stimulate IL-1β secretion [152]. Additionally, double-stranded RNA, through interaction with the Toll-like receptor 3 (TLR3), resulted in NF-κB activation [153] and was suggested to activate the NLRP3 inflammasome and IL-1β secretion [150]. Furthermore, activation of NLRP3 by double-stranded RNA has been reported, involving caspase-8 scaffolding function [154]. Similarly, a recent study on CSA-AKI in children suggested an upregulation of TLR3 and NF-κB protein in peripheral blood mononuclear cells (PBMCs), and IL-1β protein in the according serum samples [155].

Increased urinary concentrations of IL-18 protein have been reported in CSA-AKI. The pattern of changes in urinary IL-18 concentration in CSA-AKI is heterogeneous, due to different timing of the sample collection in relation to the operation period/use of CPB, differences in patient populations, and probably also due to differences in patient management. The current data suggest that timing is an important aspect. Studies have so far described one protein concentration peak either approximately 0–6 h after operation [156,157] or 12–18 h after CPB [158,159]. Other studies reported two peaks (approximately 0–6 h and 12–18 h after operation) [160] or a broad peak at these points [161] in paediatric patients. The picture is complicated by the fact that non-AKI patients often show a similar pattern of urinary IL-18, so that the difference between AKI and non-AKI is quantitative, with lower IL-18 concentrations in non-AKI patients at one or several points in time [156,159,160,161,162]. A higher resolution of the temporal pattern of IL-18 concentrations, especially with respect to the start of rises in concentration, and a direct investigation of involved inflammasome components could promote the understanding of CSA-AKI pathogenesis.

#### 3.2.2. Cardiorenal Syndromes (CRS)

Cardiorenal syndrome refers to the bidirectional nature of interactions between dysfunction of the heart and dysfunction of the kidneys, and has been classified into five types [163]. 

In CRS type 1, acute heart failure results in AKI. The underlying pathophysiology involves hemodynamic and non-hemodynamic mechanisms, including oxidative stress and inflammation [164]. Plasma from patients with CRS type 1, compared to healthy control plasma, induced higher caspase-8 activity in monocytes [165] and caspase-3, -8, -9 activity in renal tubular epithelial cells [166]. Plasma from patients with CRS type 5, where cardiac and renal dysfunction are secondary to the same systemic disorder, induced caspase-3, -8, -9 activity and cell death in renal tubular epithelial cells [167]. In CRS type 1, increased serum concentrations of IL-18 protein were reported [168] and increased plasma IL-1β in CRS type 5 [167]. Inflammasome-mediated non-canonical caspase-8 activation that contributes to IL-1β production has been described [169], but also, in mitochondrial apoptosis, the parallel induction of caspase-8 and the NLRP3 inflammasome, both contributing to IL-1β production [170]. Hence, the involvement of inflammasome components in the pathogenesis of CRS is possible, but has not been directly addressed so far in humans.

#### 3.2.3. Sepsis-Associated AKI (S-AKI)

In S-AKI, the acute reduction in kidney function is associated with infection or sepsis. Several DAMPs that could be involved in human S-AKI have been described [171]. DAMPs contribute to inflammasome priming by increasing the gene expression of inflammasome components, as NLRP3, and inflammasome substrates, like pro-IL-1β [10,11]. This review did not identify direct analyses of inflammasome components in human S-AKI. Increased concentrations of serum IL-1β protein concentration in S-AKI have been reported [172,173]. On the other hand, elevations of IL-1β serum concentrations in human sepsis can be mild [174] or irregular [175], are dependent on the phase of septic state [176], and, in addition, the kidney has a role in IL-1β removal in sepsis as long as diuresis is preserved [177]. Recently, a study reported the association of urinary IL-18 protein concentrations with AKI progression in critically ill patients at the intensive care unit [178] and a meta-analysis of biomarkers in S-AKI reported a correlation of urinary IL-18 and the diagnosis of S-AKI [179].

Serum NLRP3 protein concentrations have been reported in septic patients [180]. The NLRP3 concentrations in this study were significantly higher in patients with septic shock compared to healthy controls, intensive care unit controls, and septic non-shock controls. Higher NLRP3 serum concentrations were associated with higher 30-day mortality. The relation to renal function, hence to S-AKI, was not specifically analysed. Thus, mechanisms of IL-1β and IL-18 elevations and inflammasome-related pathomechanisms in human S-AKI require further study.

#### 3.2.4. Contrast-Induced AKI (CI-AKI)

In CI-AKI, a reduction in kidney function develops after administration of iodinated contrast material. The underlying pathogenesis is multifactorial. Upon injection of iodinated contrast, medullary ischemia and tubular cell toxicity can develop [60]. The generation of reactive oxygen species and the activation of subsequent signalling pathways, as well as the development of oxidative distress, resulting in inflammation and increased apoptosis, have frequently been reported and contribute to kidney function impairment [181].

The involvement of NLRP3 in CI-AKI was recently described [60]. The suggested mechanisms involve contrast-induced activation of the canonical NLRP3 inflammasome in renal macrophages and subsequent IL-1-dependent leukocyte recruitment. The clinical part of the study showed that urinary caspase-1protein was associated with leukocyturia. An increase of urinary IL-18 protein and the tubular injury marker kidney injury molecule-1 (KIM-1) was observed in the period of 12 to 24 h following contrast injection. Urinary IL-1β increase was not observed, and IL-18 and KIM-1 did not predict AKI within 7 days in this cohort. The timing seems to be important in the pathophysiology of CI-AKI. A study in a patient cohort undergoing coronary angiography, reported significant increases for urinary IL-18 and KIM-1 at an earlier time point (6 h post-intervention), which predicted CI-AKI within 2 days [182]. When analysed even earlier, at 2–4 h post-intervention, changes of plasma and urinary IL-18 and KIM-1 concentrations were not significantly different between patients with and without CI-AKI within 3–5 days [183]. Taken together, inflammasome components seem to be part of the multistep/multifactorial pathogenesis of CI-AKI. Further evaluation of the temporal pattern of the underlying pathomechanisms will inform research on the timing and duration of therapeutic and prophylactic measures for CI-AKI.

#### 3.2.5. Further Data on the Involvement of Inflammasome Components in AKI

The gene expression of NLRP6 was reported in human whole-kidney samples [184]. Recently, it was shown in murine nephrotoxic AKI that NLRP6 reduced sterile inflammation and exerted nephroprotective effects [185]. This publication also reported NLRP6 protein expression in human kidney tubules. Based on the comparison of the renal immunohistochemistry between a patient with histologic acute tubular injury and clinical signs of renal function impairment and a healthy control subject, a clinical significance of tubular NLRP6 reduction in human AKI was suggested.

Another publication analysed inflammasome components in a patient with non-traumatic rhabdomyolysis-induced AKI [186]. The kidney biopsy showed tubular injury at the sites of myoglobin and uric acid deposits. Using quantitative immunohistochemistry, the authors showed associated tubular oxidative stress, inflammation and necroptosis. Adjacent to the sites of tubular injury, tubulointerstitial inflammatory infiltrates and increased staining for adaptor protein ASC and active caspase-1 were reported, pointing to inflammasome activation.

#### 3.2.6. Acute Kidney Allograft Dysfunction

##### Immediate Post-Transplantation

Reperfusion injury or postischemic ATN is the most common cause of delayed graft function (DGF) in kidney transplantation. An involvement of inflammasome components in IRI in the human kidney has been suggested. In kidney cortical biopsy samples, an enrichment of the inflammasome pathway (NLRC4 gene) under reperfusion conditions compared to the preceding ischemic phase was reported [187]. Another study suggested inflammasome activation and subsequent recruitment of IFN gamma effector cells in DGF, and reported significant upregulation of NLRC4 gene expression in pre-transplant donor kidney biopsies [188]. It is interesting that IL-18 concentrations in kidney donor urine at kidney procurement did neither correlate with the severity of acute tubular injury at the time of procurement nor with the kidney recipient-estimated glomerular filtration rate (eGFR) at six months post-transplantation [189]. On the other hand, IL-18 concentrations in recipient urine were already considerably increased directly after the surgical procedure, and continued to be high until the second post-operative day. These IL-18 concentrations were significantly higher in patients with delayed graft function compared to patients with immediate graft function and with a predicted need for dialysis within the first week after transplantation, as well as the recovery of kidney graft function at three months [190]. Together, these data point to an involvement of inflammasome components during kidney ischemia and/or reperfusion. This could be in line with results from a recent study that tested the application of multipotent adult progenitor cells (MAPCs) to reduce IRI during normothermic machine perfusion of donor kidneys. Treatment with MAPCs resulted in improved surrogate parameters for kidney function and a downregulation of IL-1β protein in kidney perfusate [191]. Therefore, interventions to modulate inflammasome activation during IRI could reduce AKI in the immediate peri-transplantation period and may improve long-term kidney transplant function, but more detailed knowledge on the nature and temporal pattern of inflammasome components’ involvement is mandatory.

The risk for IRI and DGF is higher in the transplantation of extended criteria donor (ECD) kidneys, which comprises kidneys from donors of higher age and a history of reduced kidney function or hypertension (for more details, see [192,193]). The activation of inflammasome-related pathways in ECD kidneys has recently been addressed. A comparison between ECD and non-ECD perirenal adipose tissue samples, used as surrogate for the corresponding kidney transplant, showed an upregulation of gene expression in the NOD-like receptor and the NFκB-signalling pathways. Gene expression of IL-1β was significantly higher in ECD renal tissue [194]. Another study found a significantly increased gene expression of NLRP3, caspase-1 and IL-1β in ECD compared to non-ECD kidney biopsies obtained immediately before kidney implantation [195].

##### Early or Late Post-Transplantation

Kidney allograft dysfunction in the early post-transplantation period is frequently caused by acute rejection of the kidney transplant. Acute T-cell-mediated rejection (TCMR) is one of the principal histologic forms of acute rejection, and an involvement of inflammasome components has been discussed [196]. Microarray analyses in a large set of dysfunctional kidney allografts revealed an association of inflammasome activation with pure TCMR. Inflammasome-related gene transcripts with association to TCMR were caspase-1, IL-1β, and IL-18. AIM2 gene expression was highly increased compared to non-TCMR kidney biopsy samples, maybe reflecting interferon gamma-dependent induction in macrophages [197].

Viral infections can cause kidney allograft dysfunction. The DNA virus BK polyomavirus can reactivate under immunosuppression and result in BK polyomavirus-associated nephropathy in kidney transplant recipients. The pathogenesis comprises the upregulation of multiple inflammatory pathways with subsequent tubular injury, tubular atrophy, and fibrosis. An analysis comparing IL-18 in kidney biopsies between BK polyomavirus-associated nephropathy and TCMR reported significantly higher gene expression of IL-18 in BK polyomavirus-associated nephropathy. Additionally, a significantly higher IL-18 protein amount was reported, which in BK polyomavirus-associated nephropathy, could be localized to the tubular epithelium, while in TCMR, the IL-18 protein was mainly localized in CD163-positive macrophages [198]. An overview of inflammasome components involved in human AKI is given in Table 3 and Table 4.

## 4. Inflammasome Components in CKD 

### 4.1. Preclinical Data on Inflammasome Components in CKD

The NLRP3 inflammasome also contributes to the pathogenesis of CKD. In murine unilateral ureteric obstruction (UUO), IL-18 neutralization reduces kidney damage and fibrosis [199]. Additional biological factors, such as the ECM components biglycan and hyaluronan, stimulate NLRP3 inflammasome activation, thus aggravating CKD progression [200]. Biglycan-deficient animals are resistant to damage following UUO [53]. Additionally, NLRP3-deficient mice have less tubular damage and decreased renal inflammation when compared to wild-type controls upon UUO [127,201,202]. The NLRP3 inflammasome was implicated in the pathogenesis of multiple kidney diseases, such as adenine-induced tubulointerstitial nephritis [203], metabolic syndrome-associated nephropathy [204,205], hyperhomocysteinemia-induced glomerulosclerosis, proteinuria-induced tubular injury [206], angiotensin II-induced hypertensive kidney injury [207], and diabetic kidney disease (DKD) [208]. These studies demonstrated the pathological roles of NLRP3 in both inflammasome-dependent and inflammasome-independent manners during CKD. Lack of NLRP3 inflammasome and related genes results in lower kidney damage, cell death, inflammation, and fibrosis. Nevertheless, there are also still unresolved questions. For example, in UUO, blocking the renin-angiotensin system decreased NLRP3 inflammasome and boosted water channel AQP2 activity [209]. Vilaysane et al. found decreased damage in tubules, along with reduced inflammatory cell infiltration, and fibrosis in connection with a decline in caspase-1 expression, and also activated IL-1β and IL-18 upon UUO in kidney-specific, NLRP3-deficient mice [127]. The NLRP3 was found to augment the TGFβ/Smad pathway, which resulted in epithelial-mesenchymal transition and fibrosis [210]. In mice with selective elimination of NLRP3, ASC, or caspase-1, the inflammasome pathway was investigated, but no differences in glomerular disease were discovered [211]. Immune cells and resident glomerular cells, such as podocytes, endothelial cells, and mesangial cells can release IL-18 and IL-1β, which may increase the advancement of DKD [208]. Inflammasome molecules and pro-inflammatory cytokine expression levels in mice with diabetes were elevated compared to non-diabetic mice [208]. The extent of renal damage in diabetic mice was identical to wild-type mice when bone marrow from *Nlrp3*-/- and *Caspase-1*-/- mice was transplanted in db/db mice, and induction of the NLRP3 inflammasome originating from intrinsic renal cells worsened diabetic nephropathy (DN). Furthermore, hyperglycaemia activated NADPH oxidase leading to NLRP3 inflammasome activation in glomerular podocytes and subsequent podocyte damage. TXNIP antagonists and short hairpin RNA (shRNA) can stop inflammasomes from triggering DKD [212]. Therefore, decreasing NLRP3/caspase-1 production could be a potential therapy for DKD. The canonical NLRP3 inflammasome is linked to the pathogenesis of crystalline nephropathy [213]. NLRP3 inflammasomes are triggered by calcium oxalate crystals, which stimulate kidney dendritic cell activity. Subsequent, administration of IL-1β receptor inhibitors protected mice from obstructive nephropathy [214]. Furthermore, allopurinol reduced kidney inflammation by decreasing NLRP3-inflammasome activity [215]. Next, Tamm-Horsfall protein, secreted by tubular cells, serves as a platform for nanoparticles to form in diseased tubules. These nanoparticles operate as an endogenous warning signal, activating the NLRP3 inflammasome following macrophage phagocytosis [73]. The NLRP3-inflammasomes also contribute to the pathogenesis of lupus nephritis. Zhao and group discovered that decreasing NLRP3 recruitment and subsequent caspase-1 activity by blocking P2 × 7R, decreased glomerular damage in MRL/LPR mice with lupus nephritis, lowering serum anti-dsDNA antibody level, as well as IL-1β and IL-17 levels [216]. BAY 11-7082, a phosphorylated NF-κB inhibitor decreased macrophage invasion by inhibiting NLRP3-inflammasome activation resulting in decreased lupus nephritis and increased mice survival [217]. TLR7, TLR8, and TLR9 inhibitors suppress NF-κB signalling and significantly reduce the progression of glomerular damage and immune cells infiltration by decreasing the expression of renal IL-1β and NLRP3-inflammasome [203]. Subsequent studies utilizing bone marrow chimeras indicated that NLRP3 causes kidney damage and inflammation [218]. Additional treatments or medications that interfere with the signalling of the NLRP3-inflammasome could minimise or prevent tubular injury, including mitochondrial-targeted antioxidants, compound-K, neferine, allopurinol, and ghrelin [219,220,221,222]. Lu et al. found that NLRP3 gain of function mutation in myeloid cells had substantial abnormal glomerular hypercellularity and interstitial nephritis, resulting in serious albuminuria and focal segmental glomerulosclerosis, in a pristine-induced experimental lupus mouse model [223]. In lupus-prone NZM2328 mice, NLRP3 stimulation in non-myeloid cells, like podocytes, enhanced lupus nephritis progression [224]. These findings imply that NLRP3 inflammasome activation and subsequent IL-1β and IL-18 release are important drivers of lupus nephritis. Moreover, in MRL/LPR animals, inhibiting NLRP3 inflammasome activity indirectly by P2 × 7 suppression and NF-κB pathway suppressed lupus nephritis [216,217]. In oxalate-induced CKD, Knauf et al. discovered that mice fed an oxalate-rich diet showed higher NLRP3-inflammosome activation in their kidneys [218]. Mice lacking NLRP3 or ASC were reliably protected from oxalate-induced kidney fibrosis without compromising oxalate equilibrium, implying that NLRP3/ASC/caspase-1/IL-1β/IL-18 are vital factors involved in the pathophysiology of oxalate-induced CKD [18,218]. Furthermore, an IL-1β receptor inhibitor, Anakinra, treatment prevented oxalate-induced CKD in mice, implying that NLRP3 plays a role in crystalline nephropathies [18]. These findings suggest NLRP3 as a possible therapeutic candidate for type-II crystal-induced nephropathy. Likewise, pharmacological suppression of NLRP3 with CP-456,773 (also known as MCC950) and β-hydroxybutyrate rescued mice from calcium-oxalate and adenine crystal-induced kidney immune cell infiltration and interstitial fibrosis [18,225]. Additionally, animals treated with CP-456,773 were protected from adenine-induced CKD [225]. Nonetheless, CP-456,773 did not reduce NLRP3–NLRP3 or NLRP3–ASC associations, indicating an undiscovered upstream signalling mechanism that targets the NLPR3 inflammasome [226]. Notably, inhibiting NLRP3 with β-hydroxybutyrate causes a phenotypic change in macrophages, which helps to reduce kidney fibrosis [227]. 

In DKD, both immune and non-immune cells of the glomerulus released IL-1β and IL-18, which exacerbated DN [228]. Additionally, renal podocytes and endothelial cells have higher expression of NLRP3 and caspase-1, suggesting its involvement in the pathogenesis of DKD [208]. Moreover, genetic and pharmacological suppression of the NLRP3-inflammasome together with IL-1β protected mice from DKD [208,228]. Several studies also showed that allopurinol, quercetin, and saxagliptin, through reducing the NLRP3/ASC inflammasome, protected mice from DN [40,228,229,230]. Yuan et al. discovered that a lack of acid ceramidase (AC) boosted the stimulation of the NLRP3-inflammasome and the secretion of exosomes, which in turn boosted the production of IL-1β in diabetic mice [231]. Dihydroquercetin effectively protects against DN by reducing ROS-induced NLRP3-inflammasome activation [219]. Shahzad et al. conducted bone marrow transplant studies with control, *Nlrp3*-/-, and *caspase1*-/- mice and discovered that the NLRP3 inflammasome in native renal cells plays a substantial role in the evolution of DKD [208]. Notably, it is yet unknown if active caspase-1 causes pyroptosis in podocytes during DKD. Furthermore, the NLRP3 inflammasome is known to be expressed by tubular epithelial cells in the kidney [232]. As a result, the role of NLRP3 in tubules in the progression of renal interstitial fibrosis in DKD remains unknown and deserves additional investigations. The expression of the NLRP3-inflammasome in the kidney has been linked to salt-sensitive hypertension [18,225]. MCC950, an NLRP3 antagonist, lowered blood pressure and fibrosis, reduced renal inflammation, and protected against kidney failure [218]. Kidney inflammation caused by the NLRP3 inflammasome has also been linked to the advancement of IgA nephropathy. In mice, both genetic deficit and pharmacological suppression of NLRP3 employing shRNA in a preventive or therapeutic way significantly slowed the development of IgA nephropathy [233]. IL-1β receptor inhibitors additionally stopped the course of pre-existing IgA nephropathy in mice [234]. These findings imply that targeting NLRP3-inflammasome can be a promising therapeutic strategy for IgA nephropathy. It is still unclear whether NLRP3′s inflammasome-independent actions are involved in the pathogenesis of IgA nephropathy. 

Besides, ASC-deficient mice were protected from hypertensive nephrosclerosis [235], and mice lacking NLRP3 were protected from hyperhomocysteinemia-induced glomerulopathy [236] and Western diet-induced nephropathy [205]. Together, inflammasome components play a crucial role in various forms of CKD, and are therefore putative therapeutic targets for CKD.

#### 4.1.1. Preclinical Data on Redox Signalling and Inflammasome Components in CKD 

Diabetic nephropathy (DN), the principal cause of end-stage kidney disease (ESKD) worldwide, is the most common CKD associated with inflammasome activation [237]. DN is characterized by ROS overproduction, glomerular barrier expansion and thickness, cell proliferation, and tubular damage that lead to fibrosis development [238]. Concerning ROS, it induces the activation of inflammasome in DN. For instance, high glucose levels upregulate toll-like receptor 4 (TLR4), which in turn produces ROS, stimulating NLRP3, ASC, and caspase-1 activity [239]. Moreover, Ang II contributes to DN by inducing glomerular mesangial expansion, membrane basement thickening, and extracellular matrix deposition, and it has been implicated in NLRP3 activation [240]. In line with this, human mesangial cells exposed to Ang II showed upregulation of NLRP3 protein, caspase-1, and ASC, leading to NLRP3 complex activation [241]. The upregulation of Ang II prompts the stimulation of NOXs via angiotensin receptor 1 (ATR1), causing ROS overproduction and driving NLRP3 activation via ROS. In agreement with the latter, podocytes and glomeruli showed the priming of NLRP3 through ROS generated via NOXs, leading to inflammation, podocytes injury and glomerular sclerosis [86]. Moreover, in the deoxycorticosterone acetate (DOCA)/salt-induced hypertension model, the levels of the 32-residue hormone peptide ELABELA are decreased, promoting the activation of NOXs that generates ROS, and subsequently, NLRP3 inflammasome activation in the renal medulla and renal cortex (Table 5) [242]. In the immunoglobulin A (IgA)-induced nephropathy model, the levels of p47-phox are elevated, leading to ROS production and oxidative stress [115]. Moreover, in this model, the Nrf2 pathway and its target antioxidant enzymes (i.e., heme oxygenase (HO-1), NADPH dehydrogenase quinone 1 (NQO-1), GPx, glutathione S-transferase (GST), and glutamate-cysteine ligase (GCL)] are downregulated, increasing even more oxidative stress [229]. In consequence, the levels of inflammasome components are upregulated, resulting in the production of IL-18 and IL-1β [115]. Supporting the latter, the treatments that activate Nrf2 reduce O_2_^•−^ and inhibit NF-κB and NLRP3 activation [243,244]. Furthermore, the inhibition of NOXs by apocynin (APO) and DPI and gp91-phox silencing attenuates caspase-1, suggesting that NOXs are implicated in NLRP3 activation [86]. In this model, the guanine nucleotide exchange factor Vav2 was involved in NOXs assembly and activation, demonstrated by Vav2 silencing, which prevents NLRP3 activation and podocyte injury [245]. Additionally, ATR1 knockout mice stimulated with Ang II do not show inflammasome activation [246]. 

In CKD models, NOX4 is the principal isoform activated due to Ang II upregulation [247]. NOX4 is highly expressed in cellular membranes like mitochondria and ER, and essentially produces H_2_O_2_ through histidine groups in its E-loop that permits the rapid dismutation of O_2_^•−^ to H_2_O_2_ [248,249]. Besides Ang II, the binding of advanced glycation end products (AGES) to their receptor, named the receptor for advanced glycation end products (RAGE), induces NOX4 upregulation in DN, which generates ROS overproduction [250]. In DN, the overproduction of ROS by AGES induces pyroptosis by activating NLRP3 and gasdermin (GSDM); the latter is an executor protein functioning as a caspase-1 substrate implicated in pore formation during pyroptosis [251]. The treatment with Tangshen formula, a Chinese herbal medicine, avoids pyroptosis by inhibiting ROS overproduction under high glucose levels, suggesting that AGES-induced ROS participates in inflammasome-mediated pyroptosis in DN [251]. 

In streptozotocin (STZ)-induced DN, the upregulation of NOX4 stimulates MAPK signalling by inducing the phosphorylation of p38 [252,253]. In accordance with the latter, the downregulation of NOX4 by small-interfering RNA and inhibition of NOX4 activity by NOX4 Inhibitor, GK-136901, lessens high glucose-induced NOXs-derived ROS generation and p38 activation [254]. The activation of p38 promotes NF-κB activation to induce the secretion of IL-1β and IL-18 expression mediated by NLRP3 activation [252]. Thus, the priming steps of NLRP3 involving ROS are present in CKD. In line with this, albumin overload induces the synthesis of chemokines such as ‘regulated upon activation, normal T cell expressed and presumably secreted’ (RANTES) and monocyte chemoattracted protein (MCP-1) through NF-κB, triggering the recruitment of monocytes and T cells mediated by the secretion IL-1β and IL-18. The latter attracts neutrophils and molecules that further generate ROS accumulation [241]. Albumin is considered a DAMP able to trigger inflammasome priming because the levels of NLRP3 are albumin dose-dependent [255]. Concerning NF-κB, several authors have suggested a crosstalk between NF-κB and Nrf2, where NF-κB activation prompts Nrf2 inhibition, leading to the transcription of inflammatory genes and preventing antioxidant gene response [256,257]. In line with this, the activation of Nrf2 and its targets genes such as HO-1 and NOQ1 ameliorates kidney damage by decreasing the p65 NF-κB subunit along with the priming of NLRP3 [258,259]. In contrast, Nrf2 deletion does not inhibit NLRP3, TXNIP, IL-1β, Il-18 and cleaved caspase-1 upregulation, suggesting that Nrf2 is needed to avoid inflammasome priming [259]. Moreover, the enhancement of Nrf2 levels decreases apoptosis, attributed to NLPR3 inflammasome blocking [260]. Another protein that activates NLRP3 is never in mitosis gene A (NIMA)-related kinase 7 (NEK7) [261]. NEK7 participates in NLRP3 complex assembly, acting as a selective regulator of NLRP3 [262]. In this sense, the in vitro model uric acid (UA)-induced urolithiasis shows that UA causes ROS overproduction, which activates NEK7, triggering NLRP3 complex assembly [261]. 

#### 4.1.2. Preclinical Data on Mitochondria and Inflammasome Components in CKD

Several authors have suggested that Ang II is implicated in maintaining mitochondrial homeostasis in CKD; however, the upregulation of Ang II during pathological conditions like DN causes mitochondrial dysfunction [263,264]. The latter is due to the stimulation of NOXs by Ang II, which induces crosstalk with mitochondria, causing mtROS overproduction and inducing mitochondrial damage [263]. AGES also generates mtROS by establishing mitochondrial crosstalk [247]. Mitochondrial ROS are implicated in tubular damage, either activating or deactivating redox-sensitive proteins. For instance, high levels of albumin generate mtROS, leading to activation of NLRP3 priming via stimulation of NF-κB [255]. In DN, mtROS also induces inflammasome priming steps by inducing the expression of TXNIP, decreasing TRX levels and promoting NLRP3/IL-1β and TGFβ1 expression [265]. In addition, the treatment of HK-2 cells with mitochondrial-targeting antioxidants coenzyme (mitoQ) impeded the dissociation of TRX from TXNIP and blocked the interaction between NLRP3 and TXNIP, highlighting the involvement of mtROS in priming NLPR3 activation [265]. The silencing of TXNIP by RNA interference triggers NLRP3 downregulation and inhibits IL-18 and IL-1β release in high-glucose environments [266]. 

The activation of inflammasomes has been related to fibrosis development because mtROS induces upregulation of the fibrosis master TGFβ1 [267]. In DN, the knockout of the NLPR3 gene decreases fibrosis by reducing fibronectin, collagen I and collagen IV along with downregulation of TGFβ1, Smad2, 3, and connective tissue growth factor (CTGF), improving tubulointerstitial fibrosis and preventing glomerular basement membrane thickness [268]. The mechanism involved in fibrosis inhibition is NLRP3 inflammasome-dependent, suggesting that NLRP3 activation is needed to mediate fibrosis in DN [268]. Furthermore, the activation of TGFβ1 leads to epithelial-mesenchymal transition (EMT), and NLRP3 has been implicated in this process because NLRP3 promotes the upregulation of α-SMA, E-cadherin expression and the induction of myofibroblasts [269]. In this context, Song et al. [270] reported that NLRP3 silencing avoids EMT under high glucose levels by blocking TGFβ1. Additionally, the authors demonstrated that in vitro, the treatment of HK-2 cells with TGFβ1 generates ROS and EMT. Interestingly, the use of NAC decreases both, suggesting that TGFβ1 generates ROS. According to the latter, mtROS are generated through TGFβ1 by establishing crosstalk with mitochondria in CKD models like UUO [271]. 

Several authors have attributed that fibrosis induction via NLRP3 is independent of inflammasome activation [17]. For instance, hypoxia induces NLRP3 upregulation independent of ASC, caspase-1, and IL-1β [87]. Consequently, NLRP3 relocalizes in the mitochondria and colocalizes with MAVS, causing ROS production and mitochondrial membrane depolarization without inducing inflammasome activation [87]. The NLRP3 interaction with MAVS induces tubulointerstitial fibrosis, emphasizing the independent mechanisms of NLRP3-induced fibrosis through MAVS [87]. 

Previous reports in DN showed that the increase of triglycerides, inducing lipotoxicity and later kidney damage is related to fibrosis onset [272]. In this regard, the activation of NLRP3 also promotes lipid accumulation, as was recently demonstrated by Wu et al. [273]. These authors reported that diabetic mice increased lipid deposition via IL-1β/ROS/NF-κB p65, triggering NLRP3 priming, which induces the upregulation of sterol regulatory element-binding protein 1 (SREBP1) and 2 (SREBP2). The latter proteins are involved in lipid biosynthesis. Moreover, the ATP-binding cassette transporter (ABCA1), a protein implicated in the transport of phospholipids, cholesterol, and other metabolites from cells to lipid-depleted HDL apolipoproteins, decreases [273,274]. The knockout of NLPR3 avoids inflammation and fibrosis by reducing lipid deposition, suggesting the involvement of NLPR3 to induce lipid accumulation [273]. Moreover, lipid deposition via inflammasome activation has been attributed to the upregulation of IL-1β, which stimulates the cluster of differentiation 36 (CD36) to mediate fatty acid uptake [275,276]. 

#### 4.1.3. Preclinical Data on Endoplasmic Reticulum Stress and Inflammasome Components in CKD

ER stress is essential in the development of CKD [277]. The latter is sustained since the blocking of ER stress prevents fibrosis in several CKD models [277,278]. In UUO, the chemical chaperon sodium 4-phenylbutyrate (4-PBA) decreases GRP78, CHOP, and ATF4 levels. Moreover, 4-PBA restores the spliced XBP1, ameliorating UPR in the obstructed kidney, and decreases fibrosis by downregulating TGFβ1 and its targets α-SMA and CTGF, reducing tubulointerstitial fibrosis [278]. ER stress also promotes fibrosis by activating NLRP3 inflammasome [277]. In line with this, 14 days after obstruction, ER stress activates NLRP3 to induce the upregulation of fibrosis markers α-SMA and collagen I. The activation of NLRP3 mediated by ER stress was demonstrated because the use of ghrelin, a brain peptide that acts against renal injuries, could decrease fibrosis by inhibiting NLRP3 activation and ER stress [279]. Furthermore, it has recently been reported that endothelin (ET) promotes inflammasome NLRP3 activation [280]. ET1 and ET2 belong to ET family members, which form the pathway involving ET-1 itself, endothelin-converting enzymes (ECEs) that activate ET-1, and the endothelin A receptor (ETA), and they are commonly activated in CKD, inducing cell growth, vasoconstriction, and inflammation. In vitro, HK-2 cells treated with ET-1 and ET-2 at different doses (50–200 nM) activated the UPR through upregulation of IRE1α, cleaved ATF6 and phosphorylated elF2α, along with NLPRP3, ASC and cleaved caspase-1 to both ET-1 as ET-2, suggesting that the ET pathway might prompt NLRP3 via ER stress [281]. The latter is supported because the use of phosphoramidon, an ECE inhibitor, attenuates ER stress, and later, NLRP3 priming by triggering mitophagy. Together, these results suggest that ER stress is an NLRP3 activator in CKD models.

**Figure 2:**
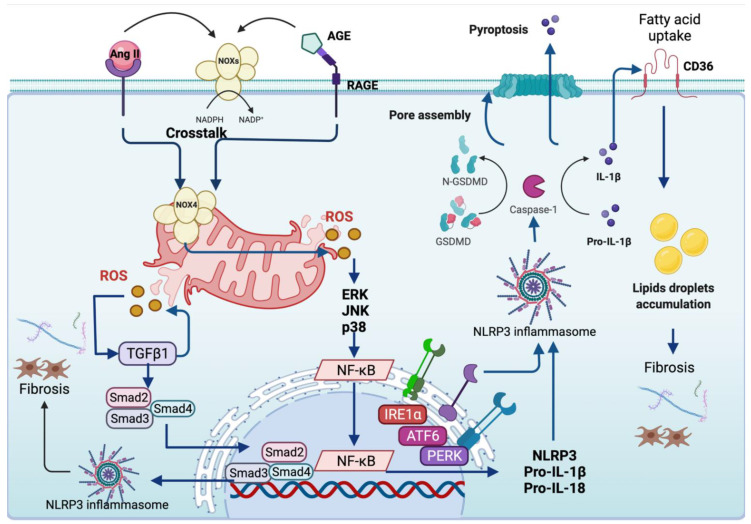
Activation of NOD-like receptor (NLR) family pyrin domain containing 3 (NLRP3) inflammasome mediated by redox-signaling, mitochondria, and ER-stress during chronic kidney disease (CKD). The stimulation of NOXs by angiotensin II (Ang II) and the advanced glycation end products (AGE) generate crosstalk with the mitochondria, inducing ROS overproduction. These mechanisms are mediated by the interaction of Ang II and AGE with their receptors: angiotensin receptor 1 (ATR1) and AGE receptor (RAGE). ROS overproduction triggers activation of extracellular signal-regulated kinase (ERK), c-Jun N-terminal kinases (JNK) and p38, which activate nuclear factor κ-light-chain-enhancer of activated B cells (NF-κB), promoting the transcription of NLRP3, interleukin 1 β (IL-1β), and interleukin 18 (IL-18) to start inflammasome activation. Moreover, ROS induces transforming growth factor-β 1 (TGFβ1) activation, triggering the formation of the Smad complex, composed of Smad2, 3, and 4, which is translocated to the nucleus. In addition, TGFβ1 activation also generates ROS, promoting NLRP3 activation to induce fibrosis development. IL-1β also leads to fibrosis by causing lipid droplets accumulation, mediated by inducing the upregulation of cluster of differentiation 36 (CD36), a receptor implicated in fatty acid uptake. Thus, fatty acid uptake triggers lipid droplet formation, inducing lipotoxicity and later promoting fibrosis. Endoplasmic reticulum stress also activates NLRP3 by generating ROS. NLRP3 inflammasome induces pyroptosis in which caspase-1 cleaves pro-IL-1β to form IL-1β, the active form, and cleaves gasdermin (GSDMD) proteins to produce an N-terminal domain (GSDMD-N). Thus, GSDMD-N can oligomerize and form pores in the plasmatic membrane, triggering increased osmotic pressure, followed by cell swelling and bursting. Figure created using BioRender (Toronto, ON, Canada).

In Figure 2, we summarized the mechanisms that induce NLRP3 inflammasome activation, which include redox signalling, mitochondria, and ER stress in CKD models. 

### 4.2. Clinical Data on Inflammasome Components in CKD

Chronic kidney disease (CKD) refers to a heterogeneous group of kidney disorders and is characterized by alterations in kidney structure or function for more than three months with implications for health. According to the KDIGO guidelines, CKD is classified based on the cause, estimated glomerular filtration rate (eGFR) category, and albuminuria category [282]. Therefore, the following section summarizes the clinical data on inflammasome components according to the cause of CKD, CKD severity (e.g., kidney failure), albuminuria, and CKD-related implications for health (e.g., CKD-attributable CVD).

It should be noted that some of the pathophysiological mechanisms described for CKD, such as in Lupus nephritis or IgA nephropathy, could also be subsumed under AKI, as the clinical course can be acute in several of the described entities.

#### 4.2.1. Albuminuria

Albuminuria is the pathological occurrence of albumin in the urine. It is a marker of CKD, but it also contributes to CKD progression. Inflammasome components were proposed to mediate albumin-induced tubulointerstitial inflammation. In kidney biopsies, NLRP3 and IL-18 protein staining increased with worsening proteinuria and correlated with inflammatory cell infiltration of the renal interstitium [283]. Furthermore, children with proteinuria and diverse underlying kidney diseases showed significantly higher NLRP3 protein expression compared to healthy control subjects, and a significant correlation between tubular NLRP3 expression and severity of proteinuria [206]. In line with this finding, epithelial caspase-1, IL-1β and IL-18 protein in proximal tubules were significantly increased with more severe proteinuria in primary and secondary glomerular disease [284]. Both results from preclinical studies and the described inflammasome-related protein changes in the proximal tubule, the site of megalin-tubulin mediated albumin reabsorption, suggest a possible involvement of inflammasome components in albumin-induced renal tubular injury and CKD progression. However, a clear dissection of such albumin-related vs. underlying kidney disease-related mechanisms is still missing.

#### 4.2.2. Nephrotic Syndrome

Nephrotic syndrome can be the result of a number of kidney diseases, and is characterized by severe proteinuria (>3.5 g per day), hypoalbuminemia, hyperlipidaemia, and oedema. Inflammasome involvement has been suggested. In peripheral blood mononuclear cells (PBMCs) from patients with glucocorticoid-resistant nephrotic syndrome, NLRP3 protein co-localized with ASC protein, which could indicate inflammasome activation [285]. In this study, methylation of the NLRP3 gene promotor, which should result in decreased NLRP3 gene expression, was significantly higher in PBMCs from patients with glucocorticoid-sensitive nephrotic syndrome. Interestingly, in kidney biopsies from children with non-minimal change nephrotic syndrome, IL-1β protein was increased compared to control samples, while in minimal-change nephrotic syndrome, which is mostly glucocorticoid-sensitive, such an IL-1β protein elevation in the kidney was not observed [286].

#### 4.2.3. Diabetic Kidney Disease (DKD)

Diabetic kidney disease refers to the presence of albuminuria and/or decreased eGFR in patients with diabetes mellitus. Classically, diabetic glomerulopathy is characterized by thickening of the glomerular basement membrane, mesangial proliferation, and podocytes loss, but other glomerular lesions and tubulointerstitial disease are found in DKD. The role of inflammasome components in DKD has gained much attention. Interleukins 18 and 1β have been investigated in the blood and urine of patients with DKD.

In agreement with the inflammatory aspects in type 2 diabetes mellitus [287], relations between renal dysfunction and inflammasome components in blood were described. The serum IL-18 protein concentrations increased with progressive DKD and correlated negatively with eGFR [288]. Serum IL-18 concentrations were significantly higher compared to healthy controls and correlated with markers of podocyte and proximal tubule dysfunction [289]. Of note, the increase of serum IL-18 seemed to precede both eGFR decrease and urine IL-18 increase. In agreement with this observation, serum IL-18 protein was associated with progression of nephropathy in normoalbuminuric patients with diabetes mellitus type 2 [290]. In type 2 diabetes PBMCs, gene expression of NLRP3 and caspase-1 was higher in DKD than in non-DKD [291]. Caspase-1 gene expression was significantly and inversely associated with eGFR in this study. In the urine, significantly higher IL-18 concentrations were found in type 2 diabetes mellitus and DKD compared to healthy controls, and IL-18 correlated with markers of podocyte and proximal tubule dysfunction [289]. Here, the rise in urinary IL-18 was most prominent in patients with reduced eGFR. In accordance with this, in a study of longstanding (≥50 years) type 1 diabetes, urine IL-18 correlated negatively with GFR [292]. These diabetic patients with and without DKD had lower IL-18 concentrations than a healthy control group. The authors concluded that these selected patients who survived longstanding diabetes with only mild kidney impairment had a low inflammatory profile, which might have been advantageous. Interestingly, in a population of type 2 diabetic patients with normal or high GFR, urine IL-18 was not different from healthy controls even if macroalbuminuria was present [293]. 

In kidney biopsy analyses, increased glomerular and tubulointerstitial IL-1β protein was found in DKD compared to non-kidney disease control subjects [294], and IL-18 protein showed a stronger expression in tubular cells of diabetic patients compared to patients with minimal change nephrotic syndrome [141]. Furthermore, kidney biopsy gene expression analysis identified IL-1β as a key regulator upstream of inflammation-related gene expression, which correlated with GFR 10 years after the kidney biopsy [295]. Significantly higher NLRP3 protein was demonstrated in DKD compared to healthy renal tissue [296]. A higher NLRP3 protein amount compared to non-diabetic and non-DKD controls was also shown in DKD glomeruli (endothelial cells and podocytes) [208]. Herein, NLRP3 colocalized with cleaved caspase-1. In these patients, serum IL-1β was increased in albuminuric diabetis mellitus and correlated with albuminuria. Moreover, significantly increased NLRP3, ASC, and caspase-1 protein was found in podocytes of DKD compared to healthy renal tissue [297]. Another study showed higher renal tubular epithelial cell NLRP3 and IL-1β protein in DKD, significantly higher urinary IL-1β and IL-18, and suggested reduced optineurin-mediated mitophagy as an underlying mechanism [298]. Preclinical data on the inflammasome involvement in kidney disease point to the importance of ROS and mitochondria. In DKD, the renal NLRP3 protein amount was significantly higher compared to kidney samples from patients without diabetes [299]. In this investigation, NLRP3 protein correlated positively with tubulointerstitial damage, glomerular damage, urine protein excretion, and dihydroethidium-detectable ROS generation. Furthermore, NLRP3 protein and the presumably inflammasome activating protein TXNIP were both significantly elevated in DKD kidney biopsies [300]. In addition to NLRP3, significantly higher NLRC4 protein content in renal tubules and interstitium was also reported in DKD compared to normal renal tissue [301]. This was paralleled by an increased tissue infiltration with CD69-positive macrophages. In line with this, NLRC4 was suggested to be increased in DKD compared to healthy renal tissue, together with a decreased protein amount of the mitophagy regulators PINK1 and parkin [302].

Investigations of inflammasome component-related pathomechanisms in DKD, especially in type 2 diabetic patients, are complicated by existing comorbidities. The presence of a metabolic syndrome—or components hereof, like hypertension or obesity—is a feature in several of the above-presented studies. This makes it more complex to draw disease-specific conclusions. We also already highlighted the increased renal tissue gene expression of NLRP3 in hypertensive/vascular nephrosclerosis in the absence of diabetes [127]. Besides, the AIM2 protein amount was significantly higher in kidney samples from hypertensive nephrosclerosis and DN [303]. In this analysis, healthy renal tissue showed primarily glomerular AIM2 protein, with some staining of tubules and interstitial leukocytes. In the CKD renal tissue samples, the glomerular AIM2 protein amount did not change, but it increased in tubular epithelial cells and leukocytes in inflammatory tubulointerstitial lesions.

Furthermore, preclinical data indicate a relation between cellular lipid accumulation and inflammasome components. In kidney biopsies from obese patients with various kidney diseases, proximal tubule cells showed phospholipid accumulation and impaired autophagy that could result in inflammasome activation [304]. Bariatric surgery to obtain weight reduction in obese patients with CKD resulted, besides the improvement of all metabolic syndrome components, in a decrease of proteinuria and a correlated decrease in serum IL-1β concentrations [305].

Several inhibitory effects of sodium-glucose cotransporter 2 (SGLT2) inhibitors on inflammasome activity in diabetes mellitus with cardiovascular disease or DKD have been suggested [306,307]. Further studies will indicate whether SGLT2 inhibitors reduce inflammasome-mediated pathophysiological mechanisms.

#### 4.2.4. Immunoglobulin A (IgA) Nephropathy (IgAN)

Immunoglobulin A nephropathy is the most common cause for primary glomerulonephritis worldwide. Current understanding of the pathogenesis is based on a “multi-hit hypothesis”, comprising occurrence of galactose-deficient IgA1 (Gd-IgA1), autoantibody formation, and immune complex deposition in the glomerular mesangium [308]. The clinical manifestations of IgAN show pronounced variability and are probably influenced by both genetic and environmental factors. Inflammasome components have been investigated in that respect. Significantly more NLRP3 protein was found in IgAN kidney biopsy samples than in healthy control renal tissue. While the protein was restricted to the tubular epithelium in healthy renal tissue, IgAN samples showed stronger tubular, but also glomerular NLRP3 protein expression [309]. As fibrotic areas no longer showed NLRP3 staining, the NLRP3 gene transcripts were inversely related to higher risks for patient and CKD prognosis in progressive IgAN. Another study also reported significantly more NLRP3 protein in IgAN kidney samples compared to normal renal tissue, and confirmed the more pronounced localization in tubules compared to glomeruli [310]. Interestingly, this study reported a significantly higher glomerular NLRP3 amount in the case that proteinuria was higher than 3.5 g/day, while tubular NLRP3 was significantly higher if eGFR was below 60 mL/min/1.73 m^2^. Additionally, in these IgAN patients, NLRP3 co-localized with Gd-IgA1 and the podocyte marker podocalyxin, suggesting Gd-IgA1-related induction of inflammasome expression in podocytes. In a patient population with immunoglobulin A vasculitis (Henoch-Schönlein pupura), urinary IL-1β was associated with nephritis occurrence [311].

#### 4.2.5. Lupus Nephritis (LN)

Systemic lupus erythematosus (SLE) is a chronic autoimmune disorder. Practically any organ system can be impaired, and LN is SLE’s renal manifestation. Diverse inflammatory pathways are involved in the organ manifestations of SLE [312]. Therefore, a longstanding interest in the involved cytokines exists, and IL-1 and IL-18 have been the focus for some time in LN [313]. In LN classes IV and V, protein amounts of active caspase-1 and IL-1β in tubular cells were suggested to be higher, compared to normal renal tissue [224]. Furthermore, the authors showed that in podocytes, active caspase-1 and IL-1β protein was only found in LN IV and V, not in normal renal tissue. The same study also investigated podocytes in urine. In accordance with the biopsy results, these urinary podocytes showed active caspase-1 and IL-1β protein in urine from patients with active LN, but not in urine from healthy controls. Other researchers, who also reported increased IL-1β in LN, localized it to renal monocytes/macrophages. In the glomeruli and interstitium of LN class IV, more IL-1β protein was found compared to other glomerulonephritides [314]. Dual-staining in this analysis suggested infiltrating monocytes/macrophages as the main cytokine source. In line with this, intracellular IL-1β protein was found in immunologically active monocyte-derived macrophages in the urine of LN patients [315]. Since according monocyte-derived macrophages were also found in the tubulointerstitium of LN kidney samples, a relevance for tubulointerstitial injury in LN was suggested. In addition, a significantly higher IL-18 protein amount was reported in infiltrating mononuclear cells in LN class IV, compared to class III and V glomeruli [316]. In this study, the serum IL-18 protein concentration in LN IV was significantly higher compared to healthy control sera and correlated positively with disease activity. In relation to the results above, an investigation of PBMCs from patients with SLE and LN is interesting. Compared to healthy controls, PBMCs in SLE had significantly higher caspase-1, IL-1β and IL-18 gene and protein expression [317]. This was paralleled by significantly increased serum IL-1β and IL-18 protein concentrations. In contrast, these SLE PBMCs had significantly lower NLRP3-activating NEK7, NLRP3, and ASC gene expression, as well as significantly lower NEK7 and NLRP3 protein amounts. In PBMCs from patients with LN, the NEK7, NLRP3 and ASC gene and protein expression were even significantly lower compared to patients without LN, and caspase-1, IL-1β, and IL-18 gene and protein expression were even higher. These results could indicate a negative feedback mechanism in PBMCs that diminishes the amount of some NLRP3-related components in the case of higher IL-1β and/or IL-18 concentrations, but confirmation is required. 

In LN renal tissue, on the other hand, NLRP3 gene expression was shown to be higher than in normal control renal tissue [127]. Analyses in whole blood from patients with a median SLE disease duration of eight years did not show a difference for SLE and healthy control IL-18 gene expression. However, within the SLE patient group, IL-18 mRNA correlated positively with disease activity and was upregulated during severe flares [318]. Analyses of circulating cytokine concentrations mainly support the notion of inflammasome involvement in LN. Patients with SLE without immunosuppressive therapy had higher serum IL-1β protein concentrations compared to healthy controls [319]. In this patient collective, IL-1β concentrations in LN were nominally even higher than in non-LN SLE. In contrast, a patient population with a majority receiving immunosuppressive treatment showed no IL-1β elevation compared to healthy controls, and no association between IL-1β and renal impairment [320]. Still, serum IL-18 protein concentration in these patients was significantly associated with active LN. In children with SLE, serum IL-18 protein concentration was significantly higher compared to healthy controls, and correlated to LN activity [321]. Serum IL-18 at 6 months post-treatment predicted renal outcomes in these paediatric patients. In agreement with the results above and a possible involvement of inflammasome components in LN pathogenesis, a serum protein pattern analysis showed the usefulness of serum soluble IL-18 receptor 1 and caspase-8 for the prediction of active LN [322].

Taken together, regulation of inflammasome components is involved in LN pathophysiology. However, the exact method of inflammasome activation and the nature of the involved inflammasome sensory components, and particularly the role of circulating IL-18, requires further study in LN and SLE. 

#### 4.2.6. Autosomal Dominant Polycystic Kidney Disease (ADPKD)

Autosomal dominant polycystic kidney disease is a hereditary disease that can lead to CKD. Patients presenting with kidney cysts can also have cysts found in other organs, such as the liver or pancreas. Several mechanisms in tubular epithelial cells have been suggested to promote cyst growth, including reduction of functional polycystins, dysregulated cell proliferation, and apoptosis [323]. Apoptosis in ADPKD was described as an early event that was found mainly in normal-appearing tubules and small cysts, probably triggered by an extrinsic factor. Caspase-8 and caspase-3 protein seemed to be involved in the apoptotic pathway [324]. As multiple interconnections between caspase-8 and inflammasome action and activation have been described [8,325], inflammasome components are worth being investigated in ADPKD pathogenesis. In addition, cardiovascular events are a major cause of death in patients with ADPKD, and hypertension and overweight are risk factors for CKD progression in these patients. Inflammasome components are also of interest in this respect. Patients with ADPKD and metabolic syndrome had a more rapid loss of GFR than ADPKD patients without metabolic syndrome [326]. In these ADPKD patients with metabolic syndrome, IL-1β plasma protein was significantly higher compared to non-ADPKD, non-diabetic patients with comparable renal function. In addition, IL-1β gene expression in the whole blood of ADPKD patients was associated with hypertension [327]. This is in line with a study suggesting an association between serum NLRP3 protein concentration and endothelial dysfunction in ADPKD [328].

#### 4.2.7. Uric Acid Kidney Diseases

Three types of kidney disease can result from the renal deposition of either urate crystals or uric acid: uric acid nephrolithiasis, which is a urinary tract stone disease; acute uric acid nephropathy, which results in AKI; and chronic urate nephropathy. Molecular mechanisms of crystal-related kidney injury have been reviewed elsewhere [69]. Here we present data from patients with CKD diagnosed with urate nephropathy (UAN). Serum NLRP3 mRNA concentrations, IL-1β, and IL-18 protein concentrations were reported as significantly higher in UAN compared to healthy controls [329]. In a patient population with gout, IL-1β protein concentrations in serum were significantly higher compared to healthy controls, and patients with UAN had significantly more IL-1β than patients with gout but without renal damage [330]. 

In another investigation, the NLRP3, ASC, caspase-1 gene and protein expression in PBMCs, and the IL-1β and IL-18 protein concentrations in plasma of patients with hyperuricemia and patients with UAN were significantly higher compared to healthy controls [331]. In these analyses, the plasma IL-1β concentrations and the PBMC caspase-1 and ASC gene and protein expression were significantly higher in UAN compared to hyperuricemia patients. The PBMC gene and protein expression for NLRP3 and the plasma IL-18 protein concentration, in contrast, were not. This could suggest negative feedback or regulation of NLRP3 in PBMCs through IL-1β or uremia.

#### 4.2.8. Anti-Neutrophil Cytoplasmic Antibody (ANCA)—Associated Vasculitis (AAV)

Anti-neutrophil cytoplasmic antibody-associated vasculitis is a small-vessel vasculitis that is associated with the presence of ANCA specific for myeloperoxidase (MPO) or proteinase 3 (PR3). Renal involvement is present in the majority of patients with AAV. Inflammasome components have been investigated in AAV kidney biopsies. One study investigated specifically tubulointerstitial injury in MPO-AAV patients diagnosed with glomerulonephritis [332]. In these patients, NLRP3 and IL-1β gene expression correlated positively with the severity of tubulointerstitial injury. NLRP3 protein was detected in infiltrating macrophages, while NLRP3 protein staining was minimal in glomeruli. Another study (also mainly MPO) in AAV reported increased protein amounts of NOD2, NLRP3 and NLRC5 in both tubulointerstitium and glomeruli compared to healthy control renal tissue [333]. Protein staining of NOD2, NLRP3 and NLRC5 was mainly seen in podocytes and infiltrating monocytes/macrophages. NOD2 and NLRC5 staining was stronger in more severe (crescentic) glomerulonephritis.

#### 4.2.9. End-Stage Kidney Disease (ESKD)

Once a patient with CKD reaches an eGFR < 15 mL/min/1.73 m^2^, signs and symptoms related to the accumulation of fluid and uremic retention solutes occur. In this end-stage of kidney disease, patients require renal replacement therapy with peritoneal dialysis (PD), hemodialysis (HD), or kidney transplantation to continue life. Chronic inflammation contributes to the progression of kidney disease to ESKD [6,334]. Inflammation in ESKD is likely multifactorial and contributes to a uremic phenotype with CVD, osteoporosis, and frailty [335]. Oxidative distress contributes to the pathogenesis of CVD in ESKD [336], and CKD stage-dependent alterations of antioxidant enzymes have been reported [337,338,339].

Inflammasome components were investigated in ESKD under several aspects. A study of neointimal hyperplasia in low-flow arteriovenous fistulas from HD patients showed a significantly higher NLRP3 protein amount compared to control vessels from a patient population matched with respect to age, diabetes and hypertension [340]. The increased amount of NLRP3 was primarily localized to neointimal vascular smooth muscle cells. Caspase-1/IL-1β, as well as Smad2/3 signalling, were activated in the arteriovenous fistulas. In an investigation of serum caspase-1 protein concentrations, significantly more circulating caspase-1 was found in ESKD compared to age-matched healthy control subjects [341]. Of note, caspase-1 protein was significantly higher in ESKD without renal replacement therapy compared to both HD and PD treatment.

In PBMCs from patients with ESKD and HD therapy, gene expression of NLRP3, caspase-1, IL-1β and IL-18 was significantly higher compared to healthy controls [342]. Co-localization of NLRP3/ASC and mitochondria in HD patients was described. Interestingly, in this study, a significantly higher protein amount in HD was only reported for caspase-1 and IL-1β/-18. Another study in HD patients could be in line with these results. Herein, the plasma IL-1β protein concentrations were significantly higher compared to a non-CKD control group that included diabetic and hypertensive subjects [343]. Nevertheless, the PBMCs from these HD patients did not show a secretion of caspase-1 and IL-1β in response to canonical inflammasome stimulation. This was attributed to a low protein expression of inflammasome components, including NLRP3, due to increased concentrations of the uremic toxin indoxyl sulfate. Such a reduction of NLRP3-related components or -activity in PBMCs from ESKD in comparison to a non-healthy control group without renal function impairment would also be in agreement with other investigations. In a study that compared hemodialysis patients to age- and diabetes-matched hypertensive patients, the HD patients had a significantly lower frequency of caspase-1 protein positive monocytes and a lower IL-1β protein secretion from PBMCs [344]. This could result from high indoxyl sulfate concentrations and/or negative feedback due to high IL-1β serum concentrations in HD patients.

The interleukins-1β and 18 belong to the group of uremic retention solutes. The serum concentration of IL-1β protein is reported as ~1.5 times higher in uraemia compared to normal, and IL-18 concentration as ~1.4 times higher (The European Uremic Toxins (EUTox) Database, available online at www.uremic-toxins.org, accessed on 12 November 2021). Interleukin 1β has been classified as belonging to uremic toxins with the second-highest evidence score for toxicity [345]. Per definition is the retention of uremic solutes directly or indirectly attributable to the reduction in renal clearance [346]. However, not only passive retention, but also upregulated production via caspase-1 has been suggested [347]. A considerable body of research investigated associations between interleukins and clinical conditions in ESKD, irrespective of the mechanisms underlying a certain serum interleukin concentration. Higher concentrations of IL-1β predicted hypotensive episodes during the hemodialysis procedure in ESKD [348]. Circulating protein concentrations of IL-18 were higher in HD patients with protein-energy wasting compared to HD patients without this condition [349] and predicted major adverse cardiovascular events [350], cardiocerebral vascular events [351], and, in children, left ventricular hypertrophy [352] among ESKD patients with HD therapy. In post-myocardial infarction patients with moderate CKD and increased high-sensitivity C-reactive protein concentrations, the inhibition of IL-1β with the monoclonal antibody canakinumab reduced major adverse cardiovascular events [353].

Taken together, the inflammasome substrates IL-1β and IL-18 are increased in advanced and end-stage kidney disease and are relevant to CKD-related morbidities. Both activated production and retention occur. Therefore, meaningful investigations of the relation between inflammasome components and uraemia/ESKD require a comparison to patients with less advanced CKD stages with the same underlying kidney disease, and/or patients matched for age and co-morbidities.

An overview of the alterations or involvement of inflammasome components or inflammasome substrates in human CKD is given in Table 6.

## 5. Summary and Perspectives

In summary, we demonstrated the considerable diversity of the inflammasome components and their interactions with various molecular partners involved in kidney disease pathophysiology. 

In preclinical AKI models, canonical and non-canonical NLRP3 effects have consistently been reported, but NLRP1 could be involved, too. Reactive oxygen species contribute to inflammasome priming and activation, while mitochondria and ER stress both contribute to the activation of NLRP3 inflammasomes. Involvement of inflammasome components in AKI has been described in monocytes-macrophages, but also in renal epithelial cells. Furthermore, crosstalk between inflammasome components and cell death pathways involving caspase-8 was reported, both in leukocytes and epithelial cells in AKI models. Clinical data are consistent with an involvement of inflammasome components in AKI, but data are still sparse. In line with the systemic nature of AKI and the organ-crosstalk involved, alterations of IL-1β and IL-18, but also NLRP3 concentrations have been reported in blood plasma/serum. A protective role for NLRP6 was suggested in acute tubular injury, while NLRC4, NLRP3, and AIM2 were involved in acute loss of kidney function in transplanted kidneys. Urinary IL-18 concentrations have been under intense investigation as a biomarker in AKI. Our review identified the importance of the temporal pattern of IL-18 concentration changes that could be helpful to determine the optimal timing of inflammasome-targeting therapies in AKI.

In preclinical CKD models, involvement of NLRP3 and inflammasome substrates IL-1β and IL-18 have been reported for CKD pathogenesis. Alterations of inflammasome components were localized to immune cells, resident glomerular cells, and endothelial cells. Furthermore, a multitude of mechanisms related to redox signalling, mitochondria and ER stress are known, that contribute to NLRP3 priming and activation. Clinical data clearly point to an involvement of inflammasome components in CKD; related to CKD causes, but also CKD progression and CKD-induced morbidities, like CVD. Besides the sensory component NLRP3, alterations for AIM2, NLRC4 and NLRC5 were also reported. As several of the causes underlying CKD are of systemic nature, as in diabetes mellitus or SLE, and since advanced CKD affects the entire body, alterations of inflammasome components and substrates in CKD are not only found in renal tissue, but also in circulating and infiltrating immune cells, in whole blood, and blood plasma/serum. 

Finally, we identified differential regulation of inflammasome components that require consideration in research on the pathogenesis of human CKD. First, frequent comorbidities as hypertension and metabolic syndrome, but also treatments, exert effects on inflammasome components; hence, control groups should be matched accordingly. Second, PBMC reduction or unexpected low amounts of NLRP3 or caspase-1 were observed in some patient populations with LN, UAN, and ESKD. This could result from increased concentrations of uremic toxins, as shown for high indoxyl sulfate concentrations, or represent a negative feedback regulation of inflammasome components in circulating immune cells that needs further study.

## Figures and Tables

**Table 2:** Redox-signalling pathways-induced inflammasome activation in AKI.

AKI model	Redox-Signalling-Induced Inflammasome Activation	Effects	Reference
PQ-induced nephrotoxicity	ROS/NF-κB/DAPK/NLRP3	PQ produces ROS, activating to NF-κB and DAPK that in turn activates NLRP3	[98]
Methotrexate-induced nephrotoxicity	decreasedNrf2/ARE/HO-1 signalling and PPARγ	Methotrexate induces the decreasing of Nrf2 and PPARγ by promoting ROS, leading to antioxidant system decrease and lipid peroxidation	[99]
Ceftriaxone-induced urolithiasis	decreasedNrf2/HO-1	Ceftriaxone promotes inflammation and oxidative stress by activating NLRP3 and inducing ROS production and decreasing the antioxidant system.	[100]

NF-κB: nuclear factor κB; NLRP3: Nod-like receptor (NLR) family pyrin domain containing 3; Nrf2: nuclear factor erythroid 2-related factor 2; ROS: reactive oxygen species; PQ: paraquat; DAPK: death-associated; antioxidant response element (ARE); heme oxygenase 1 (HO-1); PPARγ: peroxisome proliferator-activated receptor-gamma; mitogen-activated protein kinases (MAPK).

**Table 3:** Inflammasome component involvement in human AKI.

Clinical Condition	Altered/Involved Inflammasome Component or Inflammasome Substrate	Investigated Tissue/Cells/Fluid	Reference
**S-AKI**	NLRP3 IL-1β IL-18	Serum Serum Urine	[180] * [172,173] [178,179]
**CSA-AKI**	IL-1β IL-18	Serum Urine	[155] [156,157,158,159,160,161,162]
**CRS**	IL-1β IL-18	Plasma Serum	[167] [168]
**CI-AKI**	Caspase-1, IL-18	Urine	[60]
**Acute tubular injury**	NLRP6	Renal tubules	[185]
**Rhabdomyolysis-induced AKI**	ASC, active caspase-1	Renal tubulointerstitium	[186]

* Septic patients (~25% with AKI), but relation to renal function not investigated. S-AKI—Sepsis-associated acute kidney injury; CSA-AKI—Cardiac surgery-associated acute kidney injury; CRS—Cardiorenal syndromes; CI-AKI—Contrast-induced acute kidney injury, IL—interleukin; ASC—apoptosis-associated speck-like protein containing a caspase-associated recruitment domain; NLRP—NOD-like receptor family pyrin domain containing; NOD—nucleotide-binding oligomerization domain.

**Table 4:** Inflammasome component involvement in acute loss of kidney function in transplanted kidneys.

Clinical Condition	Altered/Involved Inflammasome Component or Inflammasome Substrate	Investigated Tissue/Cells/Fluid	Reference
**Reperfusion injury**	NLRC4	Renal tissue	[187]
**DGF**	NLRC4 IL-18	Pretransplant donor renal tissue Transplant recipient urine day 0–2 post-transplantation	[188] [190]
**Extended vs. non-extended criteria donor kidneys**	NOD-like receptor and NFκB signalling pathways, IL-1β NLRP3, caspase-1, IL-1β	Perirenal donor adipose tissue Pretransplant donor renal tissue	[194] [195]
**Acute T cell-mediated rejection**	AIM2, caspase-1, IL-1β, IL-18 IL-18	Renal tissue CD 163-positive macrophages in renal tissue	[197] [198]
**BK polyomavirus-associated nephropathy**	IL-18	Renal tubular epithelium	[198]

NLRC—NLR family CARD domain-containing protein; IL—interleukin; NOD—nucleotide-binding oligomerization domain; NLRP—NOD-like receptor family pyrin domain containing; DGF—delayed graft function; NFκB—nuclear factor κ-light-chain-enhancer of activated B cells, AIM2—Absent in Melanoma 2; CD—cluster of differentiation; BK—abbreviation of the name of the first patient from whom the BK polyomavirus was isolated.

**Table 5:** Redox-signalling pathway-induced inflammasome activation in CKD.

CKD Model	Redox-Signalling-Induced Inflammasome Activation	Effects	Reference
**IgA-induced nephropathy**	NF-κB/NLRP3	The activation of NF-κB induces NLRP3 activation, required for progression promoted by IgA	[244]
**IgA-induced nephropathy**	decreasedNrf2/NLPR3	IgA deactivates Nrf2, increasing ROS and oxidative stress, inducing NLRP3 activation	[243]
**DOCA/salt-induced hypertension**	NOXs/ROS/NLRP3	The decreasing of ELABELA activates the NOXs/ROS/NLRP3 pathway	[242]
**Ang II-induced DN**	Ang II/ROS/NF-κB/NLRP3	Ang II induces proliferation and the biomarker expressions FN, Col IV and CTGF, inducing fibrosis.	[241]

IgA: immunoglobulin A; DOCA: deoxycorticosterone acetate; NOXs: NADPH oxidases; STZ: streptozotocin; Nrf2: nuclear factor erythroid 2-related factor 2; NF-κB: nuclear factor-κB; NLRP3: nucleotide-binding and oligomerization domain-like receptor family pyrin domain-containing 3; Ang II: angiotensin II.

**Table 6:** Inflammasome component involvement in CKD.

Clinical Condition	Altered/Involved Inflammasome Component or Inflammasome Substrate	Investigated Tissue/Cells/Fluid	Reference
Albuminuria	NLRP3 NLRP3, IL-18 Caspase-1, IL-1β, IL-18	Renal tubules Renal interstitium Proximale renal tubule epithelium	[206] [283] [284]
Nephrotic syndrome	NLRP3, ASC IL-1β	PBMCs in glucocorticoid-resistant nephrotic syndrome Renal tissue in non-minimal change nephrotic syndrome	[285] [286]
DKD	IL-1β IL-18 IL-18 NLRP3, caspase-1 IL-1β IL-18 NLRP3 NLRP3, cleaved caspase-1 NLRP3, ASC, caspase-1 NLRP3, IL-1β NLRC4 AIM2	Serum Serum Urine PBMCs Renal tissue Glomerulum, tubulointerstitium Renal tubules Renal tissue Glomerular endothelial cells, podocytes Renal podocytes Renal tubule epithelial cells Renal tubules, renal interstitium Renal tubule epithelial cells, leukocytes in tubulointerstitial lesions	[208] [288,289,290] [289,292] [291] [295] [294] [141] [296,299,300] [208] [297] [298] [301,302] [303]
DKD comorbidities Hypertension Metabolic syndrome	NLRP3 AIM2 IL-1β	Renal tissue Renal tubule epithelial cells, leukocytes in tubulointerstitial lesions Serum	[127] [303] [305]
IgAN Nephritis in immunoglobulin A vasculitis	NLRP3 IL-1β	Renal tubules and glomeruli Urine	[309,310] [311]
LN	IL-1β IL-18 IL-18 NLRP3, ASC, caspase-1, IL-1β, IL-18 IL-1β IL-18 Active caspase-1, IL-1β NLRP3	Serum Serum Whole blood PBMCs Urinary macrophages Renal monocytes-macrophages Infiltrating mononuclear cells in renal glomeruli Renal tubules and podocytes Renal tissue	[317,319] [316,317,320,321] [318] [317] [315] [314] [316] [224] [127]
ADPKD	IL-1β NLRP3	Whole blood Serum	[327] [328]
UAN	NLRP3, IL-1β, IL-18 IL-1β ASC, caspase-1	Serum Serum, plasma PBMCs	[329] [330,331] [331]
AAV	NLRP3, IL-1β NOD2, NLRP3, NLRC5	Renal tissue, mainly infiltrating macrophages Renal tissue, podocytes and infiltrating monocytes-macrophages	[332] [333]
ESKD	IL-1β IL-18 Caspase-1 NLRP3, caspase-1, IL-1β, IL-18 NLRP3, caspase-1, IL-1β	Plasma Serum Serum PBMCs Neointimal VSMCs of low-flow arteriovenous fistulas	[343,348] [349,350,351,352,353] [341,347] [342] [340]

NLRC—NLR family CARD domain-containing protein; IL—interleukin; NOD—nucleotide-binding oligomerization domain; NLRP—NOD-like receptor family pyrin domain containing; AIM2—Absent In Melanoma 2; DKD—diabetic kidney disease; PBMC—peripheral blood mononuclear cells; ASC—apoptosis-associated speck-like protein containing a caspase-associated recruitment domain; IgAN - Immunoglobulin A nephropathy; LN—Lupus nephritis; ADPKD—Autosomal dominant polycystic kidney disease; UAN—Urate nephropathy; AAV—Anti-neutrophil cytoplasmic antibody-associated vasculitis, ESKD—End-stage kidney disease, VSMC—vascular smooth muscle cell.

## Supplementary Materials

The following supporting information can be downloaded at: https://www.mdpi.com/article/10.3390/antiox11020246/s1.
