# Supplementary material for: Involvement of Inflammasome Components in Kidney Disease
Source: Antioxidants (Basel). 2022 Jan 27;11(2):246. doi: 10.3390/antiox11020246 (PMC8868482; doi:10.3390/antiox11020246)
Supplement: Supplementary file 1 [file antioxidants-11-00246-s001.zip › antioxidants-1543494-supplementary.pdf]

antioxidants

Supplementary Table S1. Overview of inflammasome components, their functions and activators.

Inflammasome component	Structure	Function	Activator	Reference
NLRP1	Contains NBD, LRR, a function to find domain (FIIND), and C-terminal CARD regions.	NLRP1 gene encodes a member of the Ced-4 family of apoptosis proteins. Diverse pathogen enzymes degrade NLRP1 leading to inflammasome activation.	*Bacillus anthracis* lethal factor	[1,2]
NLRP2	Contains an N-terminal pyrin effector domain (PYD), other components include a centrally-located nucleotide-binding and oligomerization domain (NACHT) and C-terminal LRR.	Activates CASP1 when associated with PYCARD, leading to the secretion of mature proinflammatory cytokine IL1β	Extracellular ATP	[3,4]
NLRP3	Consists of three components - NLRP3 scaffold, PYCARD adaptor (ASC), which functions as a caspase-1 activator, and caspase-1.	Initiates an inflammatory form of cell death and triggers the release of proinflammatory IL-1β and IL-18.	Bacterial and viral nucleic acids, LPS, and damage-associated molecular patterns, such as ATP, uric acid, and amyloid β peptides. Ionic flux, mitochondrial dysfunction, and the production of reactive oxygen species, and lysosomal damage have been shown to trigger its activation.	[5-7]
NLRP6	Composed of three domains. The N-terminus consists of a pyrin domain (PYD) and is considered the essential element for inflammasome assembly as it interacts with ASC. The NBD builds the central module of NLRP6 and is followed by the C-terminal LRR domain, which senses DAMPs and MAMPs.	Regulates the production of IL-18	Bacterial products, bacterial acylated lipopeptides	[8-10]
NLRP7	Has an N-terminal pyrin domain, followed by a NACHT domain, a NACHT-associated domain (NAD), and a C-terminal LRR region.	May act as a feedback regulator of caspase-1-dependent IL-1β secretion.	Bacterial acetylated lipoproteins	[11,12]
NLRP9	NLRP9 consists of an N-terminal PYD and a central NACHT domain. The NACHT domain is directly followed by a C-terminal LRR domain that can be divided into a transition and a canonical LRR.	Initiates the formation of the inflammasome polymeric complex, made of NLRP9, PYCARD, and CASP1. Recruitment of proCASP1 to the inflammasome promotes its activation and CASP1-catalyzed IL-1β and IL-18 maturation and release in the extracellular milieu.	Viral dsRNA	[13,14]
NLRP10	Only NLR lacking the putative ligand-binding leucine-rich-repeat domain.	Negative regulator of other NLR members, including NLRP3 functions in apoptosis and the immune system. Inhibits autoprocessing of CASP1, CASP1-dependent IL1β secretion, PYCARD aggregation, and PYCARD-mediated apoptosis but not apoptosis induced by FAS or BID.	C. albicans infection	[15,16]
NLRP12	Consists of an N-terminal PYD, a central NBD, and a C-terminal LRR region.	Expressed in myeloid cells and functions as a negative regulator of inflammation by suppressing both canonical and non-canonical	Acylated lipid A	[17,18]

		NFκB signaling. The *NLRP12* gene provides instructions for making a protein called monarch-1. The monarch-1 protein is involved in inhibiting inflammation.<br>Regulation of NFκB signaling, inflammasome activation, dendritic cell migration, and transcription of MHC class I genes.		
NLRC4	NLRC4 contains a common three-domain structure: an N-terminal homotypic interaction domain, a central nucleotide-binding domain, and a series of C-terminal LRRs.	The activation of NAIP proteins attracts and activates NLRC4, which in turn attracts caspase-1 either directly or indirectly through ASC, causing inflammatory responses	Bacterium's type 3 secretion system (T3SS) and flagellin	[19,20]
NLRC5	Largest NLR family member, consisting of 1 866 aa with C-terminal 27 LRRs. Sequence analysis suggests that NLRC5 is most similar to CIITA among NLR family members.	Regulation of MHC class I gene expression, inflammasome activation in response to bacterial infection through a mechanism involving heterodimerization with NLRP3.	Bacterial PAMPs and crystals	[21-23]
NLRX1	NLRX1 contains a dissimilar and uncharacterized *N*-terminal effector domain compared to other NLRs. It also has an unusual *C*-terminus, which contains seven LRRs and a three-helix bundle.	NLRX1 localizes to mitochondria and generates mitochondrial reactive oxygen species (mROS). mROS can thereby activate the NLRP3 inflammasome.<br>NLRX1 also attenuates NF-κB and inflammasome signalling.	F. nucleatum infection	[24,25]
AIM2	AIM2 consists of two domains connected through a long linker: an N-terminal PYD domain, and a C-terminal HIN-200 domain. HIN-200 region directly binds to DNA while the PYD region mediates protein-protein interaction.	Triggers the formation of inflammasomes that also contain ASC and caspase-1, and that induce the cleavage of caspase-1, the maturation ofIL-1β,IL-18, and pyroptosis.	dsDNA, exogenous DNA of bacteria (e.g., *Listeria monocytogenes*) and viruses (e.g., *Papillomavirus*), as well as endogenous DNA.	[26-29]
IFI16	Contains a pyrin domain and two copies of the HIN-200 domain.	Serves as negative regulator of AIM2 inflammasomes by inhibiting the formation of the AIM2-ASC complex.<br>Involved in DNA binding and recognition.	dsDNA	[12,27,29]
Pyrin	Pyrin is coded by the *MEFV* gene, and its mature form includes a PYD, two B-boxes, and a coiled-coil domain.	Causes various modifications (glycosylation, adenylation, ADP-ribosylation, etc.) of Rho GTPases, causing the rearrangement of the cytoskeleton and subsequent activation of pyrin inflammasomes.	*Clostridium difficile* TcdB, *Clostridium botulinum* C3, and *Vibrio parahaemolyticus* VopS proteins.	[30,31]
ASC	Two death domains (pyrin and CARD). ASC interacts with cell death executioners.	ASC is a central adaptor molecule of the inflammasome complex, *activation* of caspase-1, mediates the secretion ofIL-1β and IL-18.	Activation of NLRP3 protein recruits ASC.	[32,33]
Caspase 1	Active Caspase 1 contains two heterodimers of p20 and p10. It contains a catalytic domain with an active site that spans both the p20 and p10 subunits, as well as a noncatalytic CARD.	Activated Caspase 1 proteolytically cleaves pro IL-1β and pro-IL-18 into their active forms, IL-1β and IL-18. The active cytokines lead to a downstream inflammatory response. It also cleaves Gasdermin D into its active form, which leads to pyroptosis.	Autoactivates when it is assembled into the filamentous inflammasome co mplex by autoproteolysis into the p10 and p20 subunits.	[34,35]
Caspase 4	Caspase 4 has Asp at the P1 position. It has a preferred cleavage sequence of Tyr-Val-Ala-Asp-∣- but also cleaves at Asp-Glu-Val-Asp-∣-.	It is an inflammatory caspase that acts as an essential effector of NLRP3 inflammasome-dependent CASP1 activation and IL1β and IL18 secretion.	UVB radiation, cholera enterotoxin subunit B, and cytosolic LPS	[36,37]

Caspase 5	Caspase 5 exists as inactive proenzyme which undergoes proteolytic processing at conserved aspartic residues to produce two subunits, large and small, that dimerize to form the active enzyme.	Initiates pyroptosis, a programmed lytic cell death pathway through cleavage of Gasdermin-D: cleavage releases the N-terminal gasdermin moiety that binds to membranes and forms pores, triggering pyroptosis. Cuts CGAS during non-canonical inflammasome activation and may play a role in the regulation of antiviral innate immune activation.	Interferon-gamma and lipopolysaccharide.	[38,39]
Caspase 11	Caspase-11 contains two components, an N-terminal CARD and a C-terminal catalytic domain. The aggregation of caspase-11 is thought to promote the auto-processing and activation of caspase-11.	An inflammatory caspase plays a crucial role in the non-canonical inflammasome. Gasdermin D (GSDMD) is the primary substrate of caspase-11, and the GSDMD cleavage fragment generated is responsible for the inflammatory form of cell death, pyroptosis, via its formation of pores in the plasma membrane.	Intracellular LPS	[13,40]

NBD - Nucleotide-binding domain; LRR - leucine rich repeat; CARD - caspase activation and recruitment domains; Ced-4 - caenorhabditis elegans protein 4; PYCARD - PYD And CARD Domain Containing; CASP1 - caspase 1; IL – interleukin; ASC - apoptosis-associated speck-like protein containing a CARD; DAMPs - damage-associated molecular patterns; MAMPs - microbe- or pathogen-associated molecular patterns; NLR - NOD-like receptor; FAS - Fas cell surface death receptor; BID - BH3-interacting domain death agonist; NFκB - nuclear factor κ-light-chain-enhancer of activated B cells; MHC - major histocompatibility complex; NAIP - neuronal apoptosis inhibitory protein; CIITA - class II transactivator; AIM2 - Absent In Melanoma 2; HIN - Hematopoietic expression, interferon-inducible nature, and nuclear localization; MEFV - mediterranean fever; LPS –lipopolysaccharide; ATP - adenosine triphosphate

References

1 Vrentas, C. E., Boggiatto, P. M., Olsen, S. C., Leppla, S. H. & Moayeri, M. Characterization of the NLRP1 inflammasome response in bovine species. *Innate Immun* 26, 301-311, doi:10.1177/1753425919886649 (2020).

2 Mitchell, P. S., Sandstrom, A. & Vance, R. E. The NLRP1 inflammasome: new mechanistic insights and unresolved mysteries. *Curr Opin Immunol* 60, 37-45, doi:10.1016/j.coi.2019.04.015 (2019).

3 Rossi, M. N. *et al.* NLRP2 Regulates Proinflammatory and Antiapoptotic Responses in Proximal Tubular Epithelial Cells. *Front Cell Dev Biol* 7, 252, doi:10.3389/fcell.2019.00252 (2019).

4 Komada, T. & Muruve, D. A. The role of inflammasomes in kidney disease. *Nat Rev Nephrol* 15, 501-520, doi:10.1038/s41581-019-0158-z (2019).

5 He, Y., Hara, H. & Nunez, G. Mechanism and Regulation of NLRP3 Inflammasome Activation. *Trends Biochem Sci* 41, 1012-1021, doi:10.1016/j.tibs.2016.09.002 (2016).

6 Sharma, D. & Kanneganti, T. D. The cell biology of inflammasomes: Mechanisms of inflammasome activation and regulation. *J Cell Biol* 213, 617-629, doi:10.1083/jcb.201602089 (2016).

7 Hoseini, Z. *et al.* NLRP3 inflammasome: Its regulation and involvement in atherosclerosis. *J Cell Physiol* 233, 2116-2132, doi:10.1002/jcp.25930 (2018).

8 Seregin, S. S. *et al.* NLRP6 function in inflammatory monocytes reduces susceptibility to chemically induced intestinal injury. *Mucosal Immunol* 10, 434-445, doi:10.1038/mi.2016.55 (2017).

9 Shen, C. *et al.* Molecular mechanism for NLRP6 inflammasome assembly and activation. *Proc Natl Acad Sci U S A* 116, 2052-2057, doi:10.1073/pnas.1817221116 (2019).

10 Lamkanfi, M. & Dixit, V. M. Mechanisms and functions of inflammasomes. *Cell* 157, 1013-1022, doi:10.1016/j.cell.2014.04.007 (2014).

11 Slim, R. & Wallace, E. P. NLRP7 and the Genetics of Hydatidiform Moles: Recent Advances and New Challenges. *Front Immunol* 4, 242, doi:10.3389/fimmu.2013.00242 (2013).

12 Radian, A. D., de Almeida, L., Dorfleutner, A. & Stehlik, C. NLRP7 and related inflammasome activating pattern recognition receptors and their function in host defense and disease. *Microbes Infect* 15, 630-639, doi:10.1016/j.micinf.2013.04.001 (2013).

13 Huang, X. *et al.* Caspase-11, a specific sensor for intracellular lipopolysaccharide recognition, mediates the non-canonical inflammatory pathway of pyroptosis. *Cell Biosci* 9, 31, doi:10.1186/s13578-019-0292-0 (2019).

14 Mullins, B. & Chen, J. NLRP9 in innate immunity and inflammation. *Immunology* 162, 262-267, doi:10.1111/imm.13290 (2021).

15 Murphy N, Grehan B, Lynch MA. Glial uptake of amyloid beta induces NLRP3 inflammasome formation via cathepsin-dependent degradation of NLRP10. *Neuromolecular Med*. Mar;16(1):205-15. doi: 10.1007/s12017-013-8274-6. Epub 2013 Nov 7. PMID: 24197756 (2014).

16 Imamura, R. *et al.* Anti-inflammatory activity of PYNOD and its mechanism in humans and mice. *J Immunol* 184, 5874-5884, doi:10.4049/jimmunol.0900779 (2010).

17 Tuladhar, S. & Kanneganti, T. D. NLRP12 in innate immunity and inflammation. *Mol Aspects Med* 76, 100887, doi:10.1016/j.mam.2020.100887 (2020).

18 Tuncer, S., Fiorillo, M. T. & Sorrentino, R. The multifaceted nature of NLRP12. *J Leukoc Biol* 96, 991-1000, doi:10.1189/jlb.3RU0514-265RR (2014).

19 Krakauer, T. Inflammasomes, Autophagy, and Cell Death: The Trinity of Innate Host Defense against Intracellular Bacteria. *Mediators Inflamm* 2019, 2471215, doi:10.1155/2019/2471215 (2019).

20 Zhao, Y. & Shao, F. The NAIP-NLRC4 inflammasome in innate immune detection of bacterial flagellin and type III secretion apparatus. *Immunol Rev* 265, 85-102, doi:10.1111/imr.12293 (2015).

21 Davis, B. K. *et al.* Cutting edge: NLRC5-dependent activation of the inflammasome. *J Immunol* 186, 1333-1337, doi:10.4049/jimmunol.1003111 (2011).

22 Meissner, T. B., Li, A. & Kobayashi, K. S. NLRC5: a newly discovered MHC class I transactivator (CITA). *Microbes Infect* 14, 477-484, doi:10.1016/j.micinf.2011.12.007 (2012).

23 Zhao, Y. & Shao, F. NLRC5: a NOD-like receptor protein with many faces in immune regulation. *Cell Res* 22, 1099-1101, doi:10.1038/cr.2012.83 (2012).

24 Reubold, T. F., Hahne, G., Wohlgemuth, S. & Eschenburg, S. Crystal structure of the leucine-rich repeat domain of the NOD-like receptor NLRP1: implications for binding of muramyl dipeptide. *FEBS Lett* 588, 3327-3332, doi:10.1016/j.febslet.2014.07.017 (2014).

25 Hung, S. C. *et al.* NLRX1 modulates differentially NLRP3 inflammasome activation and NF-kappaB signaling during Fusobacterium nucleatum infection. *Microbes Infect* 20, 615-625, doi:10.1016/j.micinf.2017.09.014 (2018).

26 Gray, E. E. *et al.* The AIM2-like Receptors Are Dispensable for the Interferon Response to Intracellular DNA. *Immunity* 45, 255-266, doi:10.1016/j.immuni.2016.06.015 (2016).

27 Wang, P. H. *et al.* Inhibition of AIM2 inflammasome activation by a novel transcript isoform of IFI16. *EMBO Rep* 19, doi:10.15252/embr.201845737 (2018).

28 Yuan, B. *et al.* Inhibition of AIM2 inflammasome activation alleviates GSDMD-induced pyroptosis in early brain injury after subarachnoid haemorrhage. *Cell Death Dis* 11, 76, doi:10.1038/s41419-020-2248-z (2020).

29 Ru, H. *et al.* Structural basis for termination of AIM2-mediated signaling by p202. *Cell Res* 23, 855-858, doi:10.1038/cr.2013.52 (2013).

30 Kim, M. L. *et al.* Aberrant actin depolymerization triggers the pyrin inflammasome and autoinflammatory disease that is dependent on IL-18, not IL-1beta. *J Exp Med* 212, 927-938, doi:10.1084/jem.20142384 (2015).

31 Xu, H. *et al.* Innate immune sensing of bacterial modifications of Rho GTPases by the Pyrin inflammasome. *Nature* 513, 237-241, doi:10.1038/nature13449 (2014).

32 Yang, Y., Wang, H., Kouadir, M., Song, H. & Shi, F. Recent advances in the mechanisms of NLRP3 inflammasome activation and its inhibitors. *Cell Death Dis* 10, 128, doi:10.1038/s41419-019-1413-8 (2019).

33 Proell, M., Gerlic, M., Mace, P. D., Reed, J. C. & Riedl, S. J. The CARD plays a critical role in ASC foci formation and inflammasome signalling. *Biochem J* 449, 613-621, doi:10.1042/BJ20121198 (2013).

34 Lu, A. *et al.* Molecular basis of caspase-1 polymerization and its inhibition by a new capping mechanism. *Nat Struct Mol Biol* 23, 416-425, doi:10.1038/nsmb.3199 (2016).

35 Vince, J. E. & Silke, J. The intersection of cell death and inflammasome activation. *Cell Mol Life Sci* 73, 2349-2367, doi:10.1007/s00018-016-2205-2 (2016).

36 Blasche, S. *et al.* The E. coli effector protein NleF is a caspase inhibitor. *PLoS One* 8, e58937, doi:10.1371/journal.pone.0058937 (2013).

37 Kajiwara, Y. *et al.* A critical role for human caspase-4 in endotoxin sensitivity. *J Immunol* 193, 335-343, doi:10.4049/jimmunol.1303424 (2014).

38 Mulvihill, E. *et al.* Mechanism of membrane pore formation by human gasdermin-D. *EMBO J* 37, doi:10.15252/embj.201798321 (2018).

39 Wang, Y. *et al.* Inflammasome Activation Triggers Caspase-1-Mediated Cleavage of cGAS to Regulate Responses to DNA Virus Infection. *Immunity* 46, 393-404, doi:10.1016/j.immuni.2017.02.011 (2017).

40 Liu, M. *et al.* Crystal structure of caspase-11 CARD provides insights into caspase-11 activation. *Cell Discov* 6, 70, doi:10.1038/s41421-020-00201-w (2020).
